# MRAP mediated adipocyte differentiation by thymic mesenchymal stromal cells contributes to thymic involution

**DOI:** 10.1038/s41467-025-64973-z

**Published:** 2025-11-20

**Authors:** Dandan Wang, Xiang Fang, Yujun Deng, Xin Wen, Ousheng Liu, Junji Xu, Fudong Fan, Dongjin Wang, Yichen Han, Peter Zanvit, Sang A. Park, Wenwen Jin, Hongbo Hu, Lingyun Sun, WanJun Chen

**Affiliations:** 1 Mucosal Immunology Section, National Institute of Dental and Craniofacial Research, National Institutes of Health, Bethesda, MD USA; 2 Department of Rheumatology and Immunology, the Affiliated Drum Tower Hospital of Nanjing University Medical School, Nanjing, China; 3 Department of Emergency Medicine, the Affiliated Drum Tower Hospital of Nanjing University Medical School, Nanjing, China; 4 Center for Immunology and Hematology, Department of Biotherapy, Cancer Center and State Key Laboratory of Biotherapy, West China Hospital, Sichuan University, Chengdu, China; 5 Department of Cardiothoracic Surgery, the Affiliated Drum Tower Hospital of Nanjing University Medical School, Nanjing, China

**Keywords:** Senescence, Cell signalling, Ageing, Autoimmunity

## Abstract

Adipocyte deposition is believed to be a primary characteristic of age-related thymic involution, but the underlying cellular and molecular mechanisms remain unknown. We show here that thymic mesenchymal stromal cells (tMSCs) have a higher tendency to differentiate into adipocytes and melanocortin-2 receptor accessory protein (MRAP) is a potential driver of tMSCs adipogenesis. Furthermore, we discover that thymosin-α1 promotes MRAP expression in tMSCs through FoxO1 signaling pathway. Additionally, the proportion of tMSCs increase in older mice compared to young mice. Importantly, MRAP is also necessary for human thymic MSCs to differentiate into adipocytes when exposed to thymosin-α1. Single-cell RNA-seq analysis of human thymus revealed an accumulation of tMSCs and adipocytes during aging, indicating a strong potential for adipogenic differentiation in age-related thymic involution. Thus, we have revealed MRAP as a key factor in promoting thymic MSCs adipogenesis triggered by thymosin-α1 and FoxO1 pathway, which may serve as potential target to hinder adiposity in age-related thymic involution.

## Abstract (web summary)

Adipocyte deposition is believed to be a primary characteristic of age-related thymic involution. Here, the authors show that MRAP is a key factor in promoting thymic MSCs adipogenesis triggered by thymosin-α1 and FoxO1 pathway, which provide a new mechanism for age-related thymic involution

## Introduction

The thymus provides a unique structural, cellular, and molecular microenvironment for the development of T cells. One characteristic of human immunological aging is the progressive decrease in the production of thymic T cells. The thymus reaches its maximum size after birth, and then gradually degenerates with age, and basically turns into adipose tissue by the age of fifty^1,2^. Adipocyte deposition is considered to be the most important feature of thymus involution in elderly individuals and accelerates the deterioration of the thymus microenvironments^3^. With the thymus involution, the reduction of thymocytes and thymic epithelial cells precedes the formation of mature lipid-laden adipocytes^4^. However, the cellular and molecular mechanisms underlying adipogenic transformation of thymus remains unclear.

A common view is that the thymus involutes at adolescence, and this model is based primarily on studies showing that growth hormone (GH) and sex steroids can affect cell production in the thymus and that their concentrations change with age^5,6^. Insulin is almost always found in the medulla, and plays a crucial role as a thymic growth factor^7^. Thymosin-α1 is a naturally occurring thymic peptide first described, which found in highest concentrations in the thymus^8^. Thymosin-α1 is thought to primarily act on innate immune cells, and also on adaptive immune responses^9,10^. However, whether and how these hormones and factors contribute to the adipogenesis during the thymic involution remain largely unknown.

Current concepts of stem/stromal cell biology suggest that adult mesenchymal stromal cells (MSCs) are present in all tissues and organs and are able to sustain repair mechanisms of injured organs^11^. MSCs can differentiate into adipocytes and osteoblasts in response to adipogenic and osteoblast-forming conditions, respectively^12^. In addition, MSCs also show immunoregulatory functions in immune cells in physiological and pathological settings^13–16^. MSCs are initially identified in the bone marrows and can be also isolated from other tissues, including but not limiting to, adipose, umbilical cord and dental tissues. In the thymus, the presence of MSCs was described only recently through differentiation assay of thymic stromal cells (TSCs)^17–19^. These early results provided indirect evidence for the presence of MSCs in the thymus. However, it is unknown whether thymic MSCs (tMSCs) play any roles in thymic involution.

In this study, we have uncovered that tMSCs preferably differentiate into adipocytes dependent on melanocortin-2 receptor accessory protein (MRAP). We determined that tMSCs had unexpectedly advantage in adipogenic differentiation but hardly to differentiate into bone tissues compared to MSCs isolated from the dental pulp. We then identified and confirmed that MRAP was required for the tMSCs-mediated adipogenesis, and this MRAP-mediated adipogenesis in tMSCs was activated and driven by thymosin-α1 through FoxO1 signaling pathway. Importantly, we demonstrated that human thymic MSCs also differentiate into adipocytes in a MRAP-dependent manner. Thus, we have revealed a previously unrecognized function of MRAP in thymic MSCs mediated adipogenesis in the thymus and opened a starting point to further understand the thymic adiposity in age-related thymic involution.

## Results

### Phenotypic identification and functional characterization of thymic MSCs

**Fig. 1:**
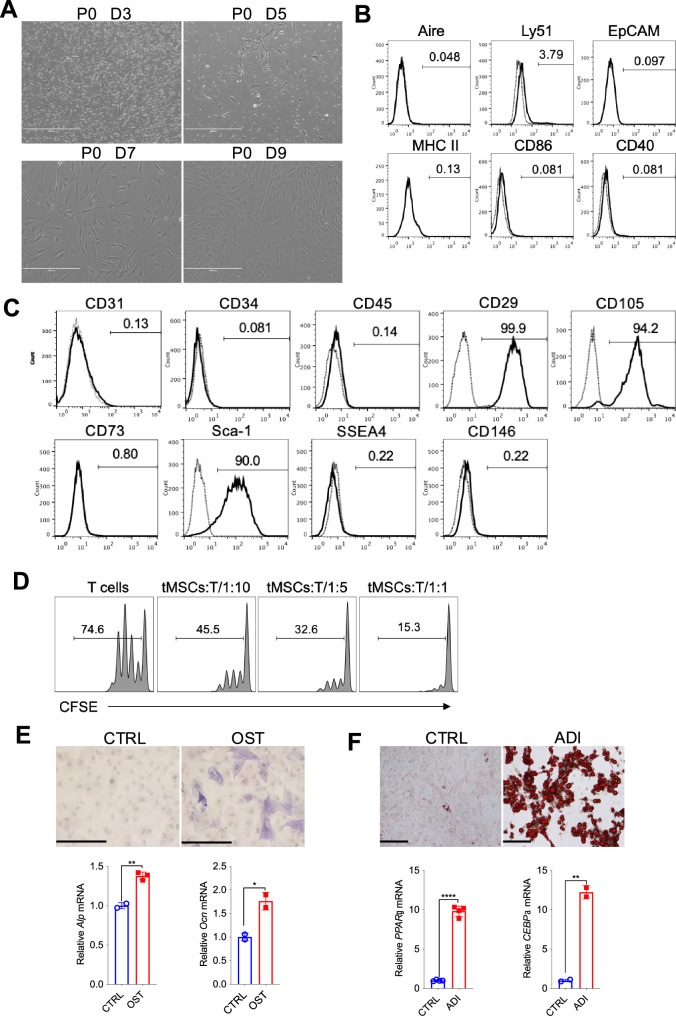
Phenotypic and functional identification of thymic MSCs. **A** Typical tMSCs in the first days (days 1–3) attaching to the culture flask, retaining their round shape. Well-spread, spindle-shaped, and adherent cells were observed in culture 3–10 days. Images are representative of three independent experiments. Scale bar, 400 µm. **B**, **C** Flow cytometric analysis of murine tMSCs with epithelial and immunological relevant antigens (**B**), and MSC-associated surface marker profile (**C**). Gray lines indicate signals for isotype matched control antibodies. **D** Flow cytometric analysis for the inhibition of T cell proliferation by tMSCs. T cells had been labeled with CFSE before the culture. The data represents two independent experiments. **E**, **F** Differentiation of tMSCs toward osteoblastic (**E**) and adipogenic (**F**) lineages, as assessed by histological staining and specific mRNA gene expression. ALP staining showing calcium deposition in osteogenic cultures. Oil red O staining showing the presence of lipid vesicles in adipocytic differentiated cells. Scale bar, 100 µm. RT-qPCR experiments are presented as the mean ± SD in differentiation cultures (black bars) as compared to control cultures (white bars) in four independent experiments. *p < 0.05, **p < 0.01 and ****p < 0.001. *p* values were determined using unpaired two-tailed t tests. Source data are provided as a Source Data file.

We firstly sought to determine whether the nonhematopoietic stromal cell population contains mesenchymal stromal cells in the thymus. We isolated the thymic stromal cell-enriched fraction from the thymus of normal adult mice by sequential enzymatic dissociation and cultures as described before with modification^20^. Thymocytes were depleted during passaging, and tMSCs displayed fibroblast-like phenotype in the culture (Fig. 1A). We next ensured the purity of thymic MSCs by excluding thymic epithelial cells (TECs) as well as thymic fibroblast that are prevalent in the stromal population but do not share an ancestry of MSCs^21,22^. Flow cytometric analysis with specific antibodies to the epithelial markers and immune cell antigens revealed that tMSCs were within the Aire^−^Ly51^−^EpCAM^−^MHCⅡ^−^CD86^−^CD40^−^ stromal cell fraction (Fig. 1B). We then examined the MSC-associated cell surface markers and revealed a pattern typical for MSCs, namely highly expressed CD29, CD105 and Sca-1, but negative for CD34, CD45, CD73, CD146 and SSEA4 (Fig. 1C). The dilution of carboxy-fluorescein diacetate succinimidyl ester (CFSE) staining assay showed that tMSCs potently suppressed T cell receptor (TCR)-driven CD4^+^ T cell proliferation in a co-culture system (Fig. 1D), consistent to the known T cell suppressive function of MSCs^23,24^. Surprisingly, when tMSCs were cultured in the osteogenic and adipogenic medium, respectively, the resulted cells were weak positive for alkaline phosphatase (Fig. 1E) but strong positive for oil red staining (Fig. 1F), respectively. Consistent with the phenotype, the expressions of osteogenic differentiated genes *Alp* and *Ocn* were hardly increased in tMSCs cultured with osteogenic medium (around 2 fold, compared to control), but the adipogenic related genes *PPARγ* and *CEBPα* were dramatically increased in tMSCs under the adipogenic culture condition (>10 fold compared to control) (Fig. 1E, F). The data suggest that tMSCs displayed stemness features but exhibited preference of adipogenic differentiation.

### tMSCs show a better adipogenesis ability

**Fig. 2:**
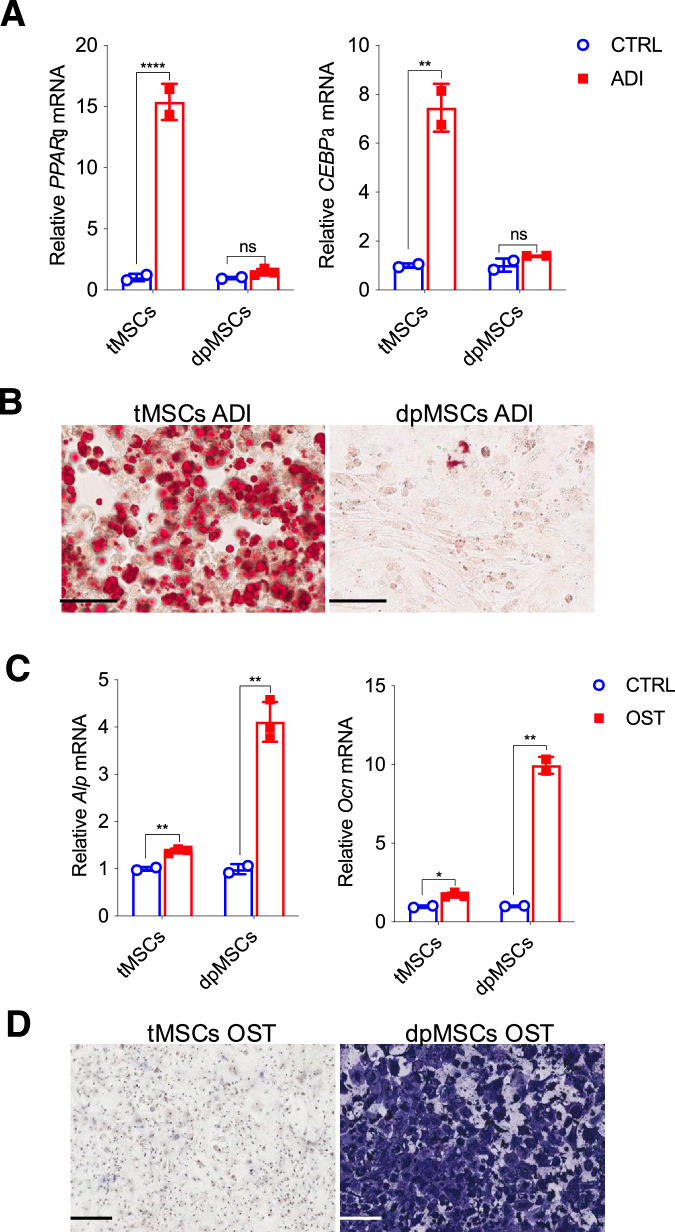
tMSCs show a better adipogenesis ability. Adipocytes (**A**, **B**) and osteoblasts (**C**, **D**) generation of both tMSCs and dpMSCs was assessed by RT-qPCR (**A**, **C**) and cytological staining (**B**, **D**), respectively. Scale bar, 100 µm. RT-qPCR experiments are presented as the mean ± SD in differentiation cultures (black bars) as compared to control cultures (white bars) in three independent experiments. **p < 0.01 and ****p < 0.001. *p* values were determined using unpaired two-tailed t tests. Source data are provided as a Source Data file.

To confirm the better adipogenic rather than osteoblastic capacity of tMSCs, we compared osteoblasts and adipocytes generated by tMSCs and dental pulp MSCs (dpMSCs) in vitro. We found that tMSCs were indeed more prone to differentiate toward adipocytes with significantly increased *PPARγ* and *CEBPα* expression (Fig. 2A) and the appearance of more lipid vesicles (Fig. 2B) during adipogenic medium, but exhibited minimal osteogenic linage in response to osteogenic stimuli (Fig. 2C, D). In contrast, dpMSCs displayed significant increase in *Alp* and *Ocn* expression (Fig. 2C), as well as calcium deposition (Fig. 2D) in osteogenic medium but showed no changes in response to adipogenic stimulation (Fig. 2A, B). The data provide compelling evidence that tMSCs intrinsically differentiate toward adipocytes.

### MRAP mediates tMSCs adipogenesis in mouse

**Fig. 3:**
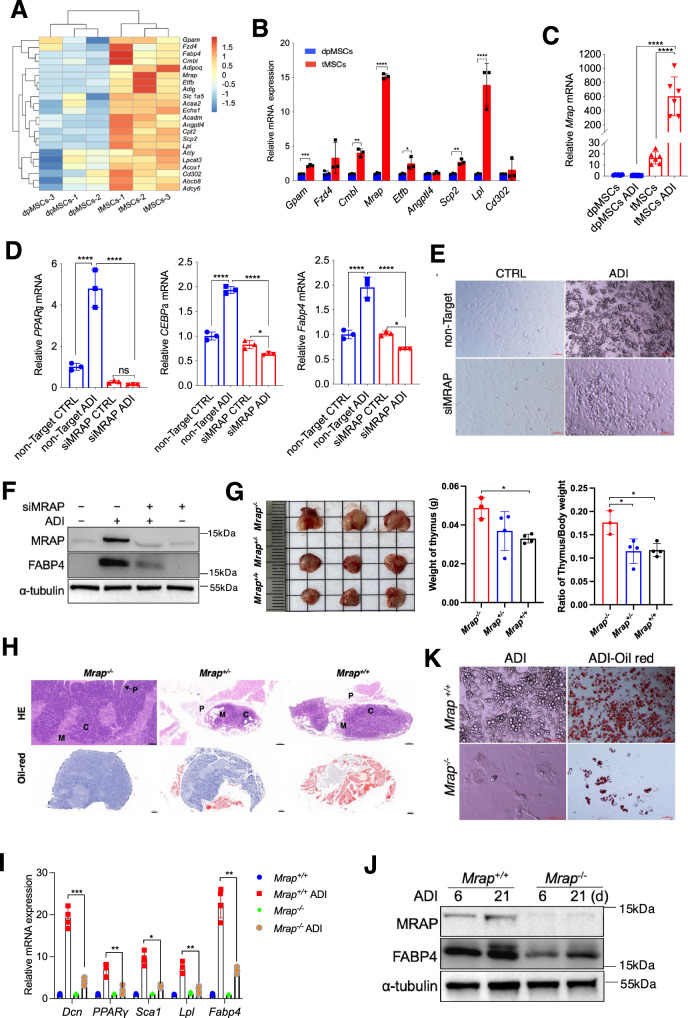
MRAP mediates tMSCs adipogenesis in mouse. **A** Cluster analysis of the genes that changed in tMSCs compared to dpMSCs (*p* < 0.05, fold change > 2). A dendrogram of the cluster correlation is present on the right. Pseudo colors show differential expression (red indicates gene levels greater than the median; white indicates gene levels equal to the median; blue indicates gene levels below the median). The heatmap was generated using log_10_-transformed reads per kilobase of transcript per million mapped reads (RPKM) values. **B** The mRNA levels of multiple members in the up-regulated genes set confirmed by RT-qPCR analysis. The data were normalized to GAPDH, and the graph represents the results of three independent experiments. **C** RT-qPCR analysis of *Mrap* mRNA levels during adipogenic differentiation in tMSCs and dpMSCs (*n* = 6 biological replicates/group). **D–F** Adipogenic related genes (*PPARγ* and *CEBPα* as well as *Fabp4*) were detected in siMRAP-transfected tMSCs by RT-qPCR (*n* = 3 biological replicates/group) (**D**). Adipogenic differentiation was evidenced by oil-red O staining (**E**) and lipid specific protein expression by Western blot (**F**). Scale bar, 100 µm. **G** Representative photographs of thymus organs in aged *Mrap*-knockout mice (7 months old) were shown (*n* = 3 mice per group, female). Thymus weight and the ratio of thymus/body weight were summarized in each group. **H** Representative histology with H&E and oil-red staining of the thymus from aged *Mrap*^*-/-*^, *Mrap*^*+/-*^ and wild-type mice (7 months old, female). *C* cortex; *M* medulla; *P* perivascular space. Scale bar, 200 µm. **I–K** Adipogenic related gene and protein expressions of tMSCs isolated from *Mrap*^-/-^ mice were analyzed by RT-qPCR (**I**) and Western blot (**J**) following adipogenic induction, respectively. Oil red O staining showing the presence of lipid vesicles in adipocytic differentiated cells (**K**). Scale bar, 100 µm. *p < 0.05, **p < 0.01, ***p < 0.005 and ****p < 0.001 (The result is representative of three independent experiments, *n* = 4 female mice per group/experiment). Data are presented as mean ± SD. *p* values were determined using unpaired two-tailed t tests: (**B**); one-way ANOVA with Tukey’s multiple-comparison test: (**C**, **D**), (**G**), (**I**). Source data are provided as a Source Data file.

Different gene expression patterns may drive the dramatic difference in adipogenesis ability between tMSCs and dpMSCs. We next isolated total RNA in MSCs purified from thymus and dental pulp (3 samples each group, from same mice) and compared their transcriptome by global RNA sequencing (RNA-seq). Unsupervised filtering of all genes according to the magnitude of regulation (SD over all 6 samples) clearly revealed different patterns of gene regulation between thymus and dental pulp derived MSCs (Fig. 3A). We confirmed these results for a selected set of genes by RT-qPCR, and nine upregulated genes were chosen in tMSCs. Of these nine genes, increased *Gpam*, *Cmbl*, *Mrap*, *Etfb*, *Scp2* and *Lpl* expression was confirmed, with *Mrap* showing the largest increase in tMSCs compared to dpMSCs (Fig. 3B). To investigate the role of *Mrap* in tMSCs-mediated adipocyte differentiation, we cultured tMSCs and dpMSCs under adipogenic differentiation condition for 3 days. As shown in Fig. 3C, the basal expression of *Mrap* was stronger in tMSCs compared to dpMSCs. Interestingly, *Mrap* expression was not increased in dpMSCs under the adipogenic differentiation condition (Fig. 3C). However, *Mrap* was significantly increased in tMSCs in response to adipogenic stimulation compared to dpMSCs (500-600 fold) and to control medium treated tMSCs (~40 fold) (Fig. 3C). To further characterize the role of MRAP in adipogenic differentiation by tMSCs, we employed a RNA interference approach to knock down MRAP expression in tMSCs and found that adipogenic genes *PPARγ*, *CEBPα* and *Fabp4*, but not osteogenic related genes *Alp* were significantly reduced in cells transfected with MRAP siRNA compared with those transfected with control siRNA (Fig. 3D and Supplementary Fig. 1). We generated large number of adipocytes from wildtype tMSCs, but much less lipid vesicles appeared in siMRAP-treated tMSCs following adipogenic differentiation (Fig. 3E). Consistently, knockdown of *Mrap* in tMSCs resulted in a marked decrease in FABP4 protein expression, which is a cytoplasmic fatty acid chaperone that expressed primarily in adipocytes (Fig. 3F). To further confirm the function of MRAP in the adipogenesis by tMSCs, we generated *Mrap* null mutation (*Mrap*^*-/-*^*)* mice. *Mrap*^*-/-*^ mice with young age appeared normal without obvious changes of body weight and the vital organs, and without signs of inflammation in the fat, kidney, liver, spleen, and lung tissues (Supplementary Fig. 2). The number of total thymocytes was not changed in young (7 weeks old) but increased in older (7 months old) *Marp*^*-/-*^ mice (Supplementary Fig. 3C). Thymocyte profile analysis showed that the *Mrap*^*-/-*^ mice exhibited higher number of CD4 single positive and double positive thymocytes at 7 months old, slightly increased number of CD8 single positive thymocytes, without changes double negative thymocytes (Supplementary Fig. 3). As we were unable to obtain old *Mrap*^*-/-*^ mice (>7 month of age), we examined the profile of T cells in the spleen, blood, and bone marrow and their phenotype such as effector/memory/naïve and Tregs in 10-month-old *Mrap*^*+/-*^ mice (Supplementary Fig. 4), and found no differences between the *Mrap*^*+/-*^ and age-matched WT mice. However, we found the older (7 months old) *Mrap*^-/-^ mice had enhanced thymic size and increased thymic weight compared with age-matched WT mice (Fig. 3G). Significantly, analysis of thymic sections from the knockout and WT mice stained with H&E and oil-red revealed that MRAP expression contributed to the dramatic disruption of thymic structure with confusing thymic medulla and cortex region and promote adipocytes deposition (Fig. 3H). We next cultured *Mrap*^*-/-*^ tMSCs under adipogenic differentiation conditions and found that the knockout tMSCs exhibited significantly reduction of the adipogenic associated genes including *Dcn, PPARγ, Sca1, Lpl*, and *Fabp4* (Fig. 3I). Consistent with siMRAP data, FABP4 protein was also substantially reduced in tMSCs of *Mrap*^*-/-*^ mice compared to wildtype mice (Fig. 3J). Consequently, a considerably reduced adipocytes formation was observed in *Mrap*^*-/-*^ tMSCs in response to adipogenic stimulation (Fig. 3K). It has been reported that MRAP deficiency impairs the production of glucocorticoid (GC)^25^. In considering the participation of glucocorticoids in thymus involution, we evaluated the GC levels in both the serum and the thymus between wildtype and *Mrap*^*-/-*^ mice, and found decreased GC levels in the serum (Supplementary Fig. 5). However, there was no difference of the GC levels in the thymus between wildtype and knockout mice (Supplementary Fig. 5). The data collectively indicate that MRAP plays a role in tMSCs adipogenesis in mouse.

### Thymosin-α1 induces MRAP expression in tMSCs

**Fig. 4:**
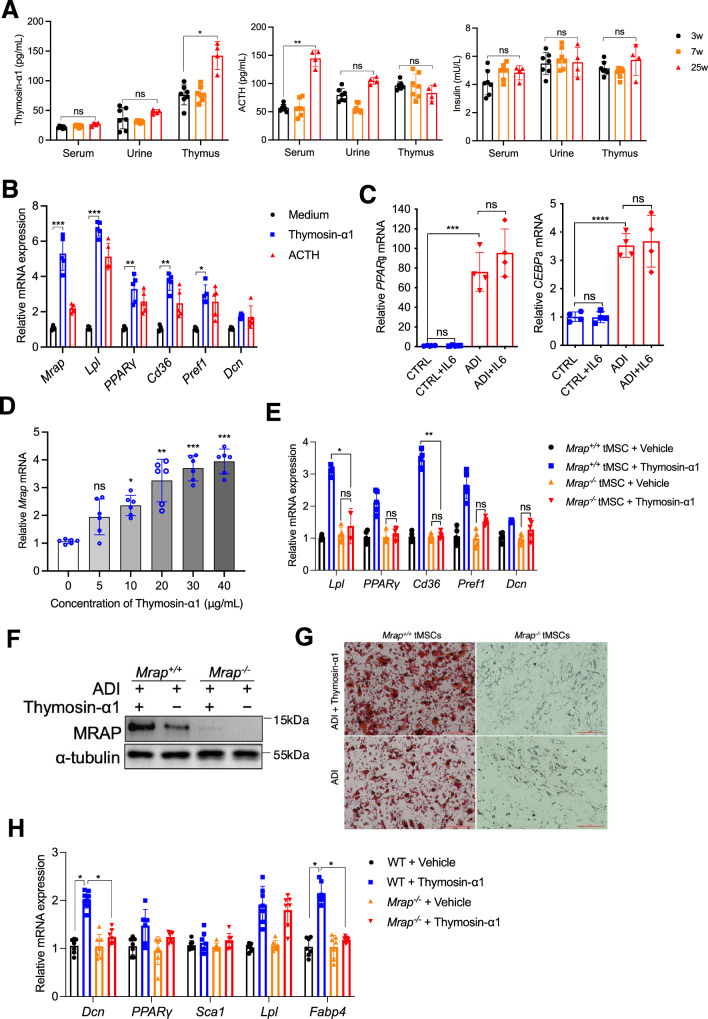
Thymosin-α1 plays a major role in MRAP expression on tMSCs. **A** Analysis of thymosin-α1, insulin and ACTH in the thymus tissue, urine and serum of mice of different ages. (The result is representative of three independent experiments, 3w/7w: *n* = 7, and 25w: *n *= 4 biological replicates). **B** Adipogenic related genes expression of tMSCs were analyzed by RT-qPCR following thymosin-α1 (50 μg/mL) and ACTH (10 nM) stimulated for 6 days (*n* = 5 biological replicates/group). **C** Recombinant IL-6 (20 ng/mL) was added in the culture system of tMSCs, alone or in adipogenic medium for 3 days, then *PPARγ* and *CEBPα* gene expression was assessed by RT-qPCR (*n* = 4 biological replicates/group). **D** RT-qPCR analysis of *Mrap* level on tMSCs following thymosin-α1 stimulation (*n* = 6 biological replicates/group). **E** Adipogenic related genes expression of tMSCs isolated from *Mrap*^-/-^ mice following thymosin-α1 (20 μg/mL) stimulated for 6 days. (The result is representative of three independent experiments, *n *= 5 female mice per group/experiment). **F**, **G** MRAP protein expression (**F**) and adipocytes formation (**G**) of tMSCs isolated from *Mrap*^-/-^ mice were analyzed following thymosin-α1 (20 μg/mL) stimulated under adipogenic differentiation culture for 21 days. (The result is representative of three independent experiments, *n* = 4 female mice per group/experiment). **H** Recombinant thymosin-α1 (1 mg/kg) were intraperitoneally transferred to *Mrap*^-/-^ and wildtype mice, thymic MSCs were isolated 14 days later and adipogenic specific genes were analyzed by RT-qPCR. *p < 0.05, **p < 0.01, ***p < 0.005 (the result is representative of three independent experiments, *n* = 7 female mice per group/experiment). Data are presented as mean ± SD. *p* values were determined using one-way ANOVA with Tukey’s multiple-comparison test: (**A**–**E**), (**H**). Source data are provided as a Source Data file.

We next investigated how MRAP was induced in tMSCs. We firstly examined the factors thymosin-α1, insulin and adrenocorticotropic hormone (ACTH), which are expressed in the thymus and associated with thymic involution in mice^5,7,10^. Analysis of these factors in the thymus, urine and serum of mice with different ages revealed that only thymosin-α1 in the thymus was significantly elevated with age (by 25 weeks), whereas ACTH was upregulated only in serum and insulin was not altered amongst the thymus, serum and urine with age (Fig. 4A). Next, we assessed the potential role of thymosin-α1 and ACTH in inducing MRAP expression and adipogenesis in cultures. We observed that thymosin-α1, but not ACTH, is the one inducing *Mrap*, although both were able to increase the expression of other adipogenesis-related genes in tMSCs (Fig. 4B). We also examined the role of IL-6, which was reported to be the only factor that increased with age, in adipogenesis of tMSCs, and the result revealed that IL-6 cannot affect the adipogenic differentiation of tMSCs (Fig. 4C). Furthermore, thymosin-α1 up-regulated *Mrap* mRNA in a dose-dependent manner (Fig. 4D). To characterize the function of thymosin-α1 in MRAP-mediated adipogenic differentiation of tMSCs, we stimulated tMSCs from *Mrap*^*-/-*^ and wildtype mice with thymosin-α1 in vitro, and showed that indeed thymosin-α1 increased adipogenic specific genes and thus had a lipid-promoting effect in the wildtype, but not *Mrap*^*-/-*^, tMSCs (Fig. 4E). We then demonstrated that thymosin-α1 indeed increased MRAP protein and adipocytes formation in wildtype, but not in *Mrap*^*-/-*^, tMSCs under the adipogenic differentiation conditions (Fig. 4F, G). Importantly, we administered recombinant mouse thymosin-α1 to *Mrap*^*-/-*^ and wildtype mice, and showed a failed upregulation of adipogenic related genes in tMSCs from *Mrap*^*-/-*^ mice (Fig. 4H). Altogether, the data reveal that thymosin-α1 is a key inducer of MRAP expression and adipogenesis by tMSCs.

### Thymosin-α1 induces MRAP and adipogenesis in tMSCs through FoxO1

**Fig. 5:**
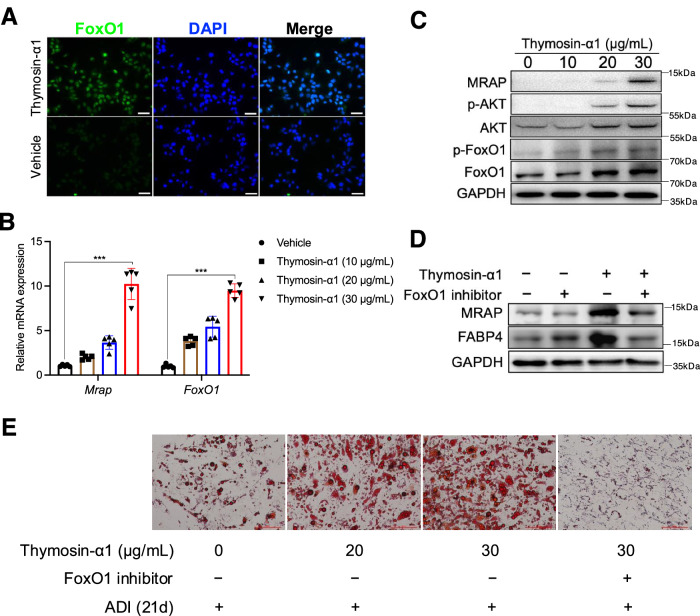
Thymosin-α1 drives MRAP mediated tMSCs adipogenesis through FoxO1 pathway. **A** Representative immunofluorescence images of purified tMSCs showing anti-FoxO1 antibodies (green) counterstained with DAPI (nuclei, blue). Data are representative of 4 independent experiments. Scale bar, 50 µm. **B** RT-qPCR analysis of *Mrap* and *FoxO1* expression following stimulated by a series of thymosin-α1 concentration to tMSCs (*n* = 5 biological replicates/group). ***p < 0.005. **C** Western blot detected MRAP and phosphorylation of AKT and FoxO1 (p-AKT and p-FoxO1) or AKT and FoxO1 in tMSCs stimulated by various doses of thymosin-α1 for 14 days. The results were from three independent experiments. **D** Thymic MSCs were stimulated with or without 20 μg/mL of thymosin-α1 in the presence or absence of FoxO1 inhibitor (2 μM) for 14 days. Cell lysates were subjected to immunoblotting analysis to detect the MRAP and FABP4. The results were from three independent experiments. **E** Thymic MSCs were stimulated with 20 or 30 μg/mL of thymosin-α1 in the presence or absence of FoxO1 inhibitor (2 μM) under adipogenic differentiation culture for 21days. Oil red O staining showing the presence of lipid vesicles in adipocytic differentiated cells (two independent experiments). Scale bar, 100 µm. Data are presented as mean ± SD. *p* values were determined using one-way ANOVA with Tukey’s multiple-comparison test: (**B**). Source data are provided as a Source Data file.

We next investigated the downstream pathway of thymosin-α1 induction of MRAP in tMSCs. It has been suggested that FoxO1 affects adipocyte differentiation by regulating lipogenesis and cell cycle^26^. Immunofluorescence studies showed that tMSCs highly expressed FoxO1 after thymosin-α1 stimulation (Fig. 5A), and RT-qPCR analysis confirmed the upregulation of *FoxO1* and *Mrap* mRNAs in tMSCs upon thymosin-α1 treatment (Fig. 5B), suggesting FoxO1 involvement in the *Mrap* expression and adipogenic differentiation of tMSCs. Western blot analysis showed that FoxO1 and its upstream phosphorylated AKT (p-AKT) were highly expressed in tMSCs in response to thymosin-α1 stimulation (Fig. 5C), suggesting that thymosin-α1 induces MRAP through AKT-FoxO1 signaling pathway. Indeed, thymosin-α1-induced MRAP and FABP4 proteins in tMSCs were markedly decreased in the presence of FoxO1 inhibitors (Fig. 5D). Consistently, thymosin-α1 induced adipocyte differentiation in tMSCs was inhibited by FoxO1 inhibitors, evidenced by the suppression of the lipid droplets formation (Fig. 5E). The data collectively indicate that thymosin-α1 drives MRAP-mediated adipocyte differentiation by tMSCs through p-AKT-FoxO1 pathway.

### Increased tMSCs number in older thymus

**Fig. 6:**
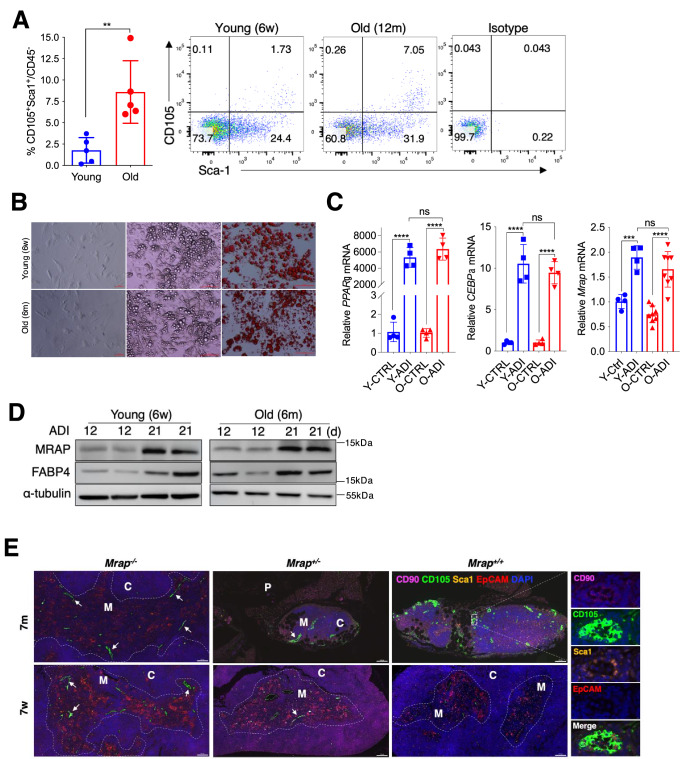
Increased tMSCs number in aged thymus. **A** Flow cytometric analysis with the composition of tMSCs in young and older mice. Representative contour plots showing the percentage of Sca1^+^CD105^+^ tMSCs among total live CD45 negative thymic stromal cells in female mice of 6 weeks old and 12 months old (*n* = 5 biological replicates/group). **B–D** Oil red O staining were compared in tMSCs derived from young and older mouse (**B**). Adipogenic related gene and protein expressions of tMSCs were analyzed by RT-qPCR (**C**) and Western blot analysis (**D**) following adipogenic differentiation, respectively. Scale bar, 100 µm. (the result is representative of four independent experiments, *n* = 4 female mice per age group/experiment). **E** Representative immunofluorescence images of thymus sections showing the distribution of tMSCs with anti-CD90 (pink), CD105 (green), Sca1 (yellow), EpCAM (red) antibodies and counterstained with DAPI (nuclei, blue). tMSCs with the character of CD90^+^CD105^+^Sca1^+^EpCAM^-^ as indicated by white arrow. *C* cortex; *M* medulla; *P* perivascular space. Scale bar, 100 µm. Data are representative of 3 independent experiments. Data are presented as mean ± SD. **p < 0.01, ***p < 0.005 and ****p < 0.001. *p* values were determined using unpaired two-tailed t tests: (**A**); one-way ANOVA with Tukey’s multiple-comparison test: (**C**). Source data are provided as a Source Data file.

Having established a critical function of thymosin-α1-FoxO1-MRAP axis in mediating adipogenesis by tMSCs, we next addressed the quantitative or qualitative changes of tMSCs in older mice. Flow cytometric analysis of the CD45^–^ stromal cells revealed that the proportion of CD105^+^Sca1^+^ tMSCs increased significantly in 12-month-old mice (older) compared to 6-week-old mice (Fig. 6A**)**. We initially hypothesized that tMSCs from older mice might possess greater adipogenic capacity than those cells in young mice. However, we found no significant difference in adipocyte-forming efficiency between young and older tMSCs under adipogenic conditions (Fig. 6B), which was confirmed by adipogenic associated genes expression (Fig. 6C). We also found that the expressions of MRAP and FABP4 were compatible between older and young tMSCs following adipogenic differentiation (Fig. 6D). The data points out that older thymus increases the relative number rather than alteration of the intrinsic ability of adipogenesis in tMSCs. Immunofluorescence analysis of thymic sections from young and older *Mrap*-knockout mice revealed that increased tMSCs (CD90^+^CD105^+^Sca1^+^EpCAM^-^) in aged WT mice, and tMSCs-rich areas in the thymus mainly run from the deep in the medullary region to the bordering the cortex regions, further confirming a critical role of MRAP in tMSCs in contributing to the thymus involution (Fig. 6E). Moreover, the frequency of tMSCs in CD45^−^ cells in young and older mice were determined (Supplementary Fig. 6). These data collectively indicate that the increase of tMSCs in aged thymus play an important role in the thymus involution through the expression of MRAP. To further confirm the correlation between thymosin-α1 and the number of tMSCs across age, we analyzed the levels of thymosin-α1 and the number of tMSCs in WT mice at different ages (Supplementary Fig. 7A and B). The results indicate that thymic thymosin-α1 increases with age and positively correlated with the number of tMSCs.

### Thymosin-α1 promotes MRAP expression and adipogenesis in human tMSCs in vitro

**Fig. 7:**
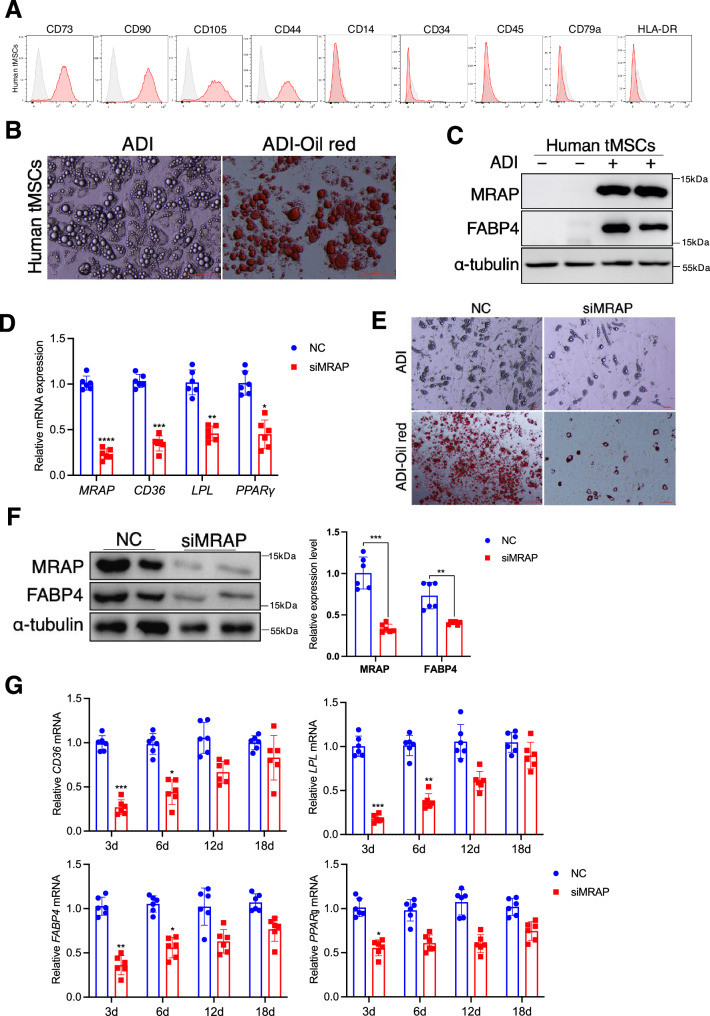
Thymosin-α1 promotes MRAP expression and adipogenesis in human tMSCs in vitro*.* **A** Representative histograms showing the expression of typical MSC markers (CD105, CD73, CD90, CD44) and absence of hematopoietic and endothelial markers (CD45, CD34, CD14, CD79a, HLA-DR) by flow cytometric analysis of human tMSCs. Gray lines indicate signals for isotype matched control antibodies. **B** Thymic MSCs were isolated from human thymus. Oil red O staining showing the presence of lipid vesicles in adipocytic differentiated cells (four independent experiments). Scale bar, 100 µm. **C** Lipid specific protein expression of human tMSCs following adipogenic differentiation were analyzed by Western blot (two independent experiments). **D–F** Adipogenic related genes (*PPARγ* and *Lpl* as well as *Cd36*) silencing appeared in siMRAP-transfected tMSCs by RT-qPCR (*n* = 6 biological replicates/group) (D). Adipogenic differentiation was evidenced by oil-red O staining (**E)** and lipid specific protein expression by Western blot (*n* = 7 biological replicates/group) (**F**) as shown. The graph represents the results of three independent experiments. Scale bar, 100 µm. **G** Adipogenic related genes expression of human tMSCs following thymosin-α1 (20 μg/mL) stimulation for 6 days (*n* = 6 biological replicates/group). Data are presented as mean ± SD. *p < 0.05, **p < 0.01, ***p < 0.005 and ****p < 0.001. *p* values were determined using unpaired two-tailed t tests: (**D**, **F**, **G**). Source data are provided as a Source Data file.

To address the clinical relevance and significance of our findings in mice, we next studied thymic MSCs isolated from human thymus that were obtained as surgical waste from patients undergoing cardiac surgery (see detailed information in Methods). Flow cytometry analysis revealed that human tMSCs exhibited the main typical MSCs surface markers (CD44, CD73, CD90, CD105) and were negative for hematopoietic (CD34), monocyte (CD14), leukocyte (HLA-DR, CD45) and B cell (CD79a) (Fig. 7A). We cultured human tMSCs (htMSCs) under adipogenic differentiation conditions and showed that they exhibited vigorous adipogenesis (Fig. 7B), similar to mouse tMSCs (Figs. 1 and 2). MRAP and FABP4 were also increased in htMSCs in response to adipogenic stimulation (Fig. 7C). Importantly, knockdown of *MRAP* with RNA interference approach resulted in significant reduction of adipogenic related genes in htMSCs (Fig. 7D) and blocked adipocyte differentiation by htMSCs (Fig. 7E). Consistently, FABP4 protein expression was substantially suppressed in siMRAP-treated htMSCs during adipogenic differentiation (Fig. 7F). Furthermore, we also cultured control- and siMRAP-treated htMSCs with thymosin-α1 under adipogenic conditions and examined the changes of adipogenic related genes in htMSCs. We found indeed knockdown of *MRAP* significantly downregulated the adipocytes associated genes expression, suggesting deficiency of adipogenic differentiation, especially at the first 3 to 6 days (Fig. 7G). Thus, human thymic MSCs also depend on MRAP to differentiate into adipocytes.

### Single cell RNA-sequencing (scRNA-seq) analysis of young and old human thymus

**Fig. 8:**
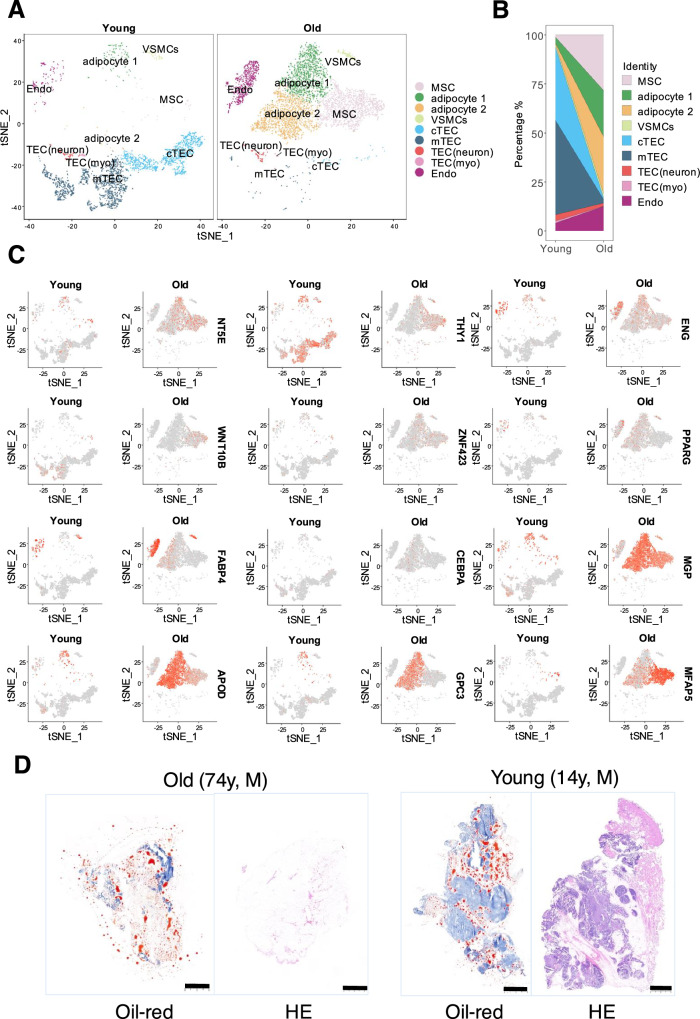
Single-cell sequencing analysis of human tMSCs and histopathology of thymus tissues. **A** UMAP plot of thymocytes from 13 subjects (8 for old group, 58.1 ± 9.7 years old; and 5 for young group 6.4 ± 4 years old) show distinct clusters predominantly determined by cell type. **B** Percentages of cell types vary between the young and old thymus. **C** UMAP plot showing the expression pattern of marker genes for MSCs, adipocyte 1 and adipocyte 2. **D** Representative histopathology with Oil-red and HE staining of the thymus tissue from male subjects with age of 74 and 14 years old (five independent experiments). Scale bar, 1000 µm for HE staining and 2000 µm for Oil-red staining.

Importantly, we applied scRNA-seq analysis on freshly isolated CD45^−^ stromal cells from the thymus tissue of 13 human subjects (8 for old group, 58.1 ± 9.7 years old; and 5 for young group, 6.4 ± 4 years old) (Supplementary Table 1 and Fig. 8). We also combined our data with other published data to increase the representation of thymic stromal cells^27^.The graph-based clustering divided the cells into 9 distinct clusters, and the differentially expressed genes (DEGs) of each cluster were identified with the Wilcoxon rank-sum test (Fig. 8A–C and Supplementary Fig. 9A). Notably, based on the cell markers, we found that a cluster expressed high levels of MSC marker genes, including *NT5E* (CD73), *THY1* (CD90), and *ENG* (CD105) as well as genes related to preadipocytes commitment including *WNT10B* and *ZNF423*^[^^28^^]^ (Fig. 8C), and this cluster increased in frequency with aging (Fig. 8B). In addition, we found that adipocyte 1 (*ZNF423* and *PPARG*) and adipocyte 2 (*PPARG*, *FABP4* and *CEBPA which* related to terminal adipocyte differentiation^28^) showed a high frequency among the old thymuses (Fig. 8C). Surprisingly, we also observed that the lipid metabolism genes (*MGP* and *APOD*)^29,30^ showed a high expression among the adipocyte clusters (Fig. 8C and Supplementary Fig. 9). Development trajectory analysis indicated that MSC developed into adipocyte 1 followed by adipocyte 2 (Supplementary Fig. 9B). Crosstalk result indicated that MSC exhibited higher extracellular matrix related crosstalk (COL1A1-ITGA5, COL1A2-ITGA11 and FN1-ITGA5) which is consistent with its stem cell identity (Supplementary Fig. 9C). Moreover, adipocyte 1 and adipocyte 2 exhibited more insulin receptor and lipoprotein receptor related crosstalk (IGF2-INSR, LPL-VLDLR and APOE-LDLR) which contribute to the regulation of energy and lipid metabolism, lipid storage, and lipid transport (Supplementary Fig. 9C). Histopathology examination also revealed a significant accumulation of adipocytes in the thymic tissue of aged individuals (Fig. 8D). Taken altogether, our findings indicate that tMSCs and adipocytes accumulate with aging and tMSCs display a strong potential of adipogenic differentiation in human thymic involution.

## Discussion

Age-related thymic involution is accompanied by loss of thymic T cells and epithelial cells and by accumulation of adipose tissues and increase in fibroblasts^2,4^. However, it remains largely unknown which types of cells differentiate into adipocytes and how the adipogenesis is regulated. It was reported that fibroblasts can be induced to develop into adipocytes and represent a cellular source of thymic adipogenesis^31,32^, but whether other types of cells in the thymus play roles remain unknown. In this study, we have discovered that thymic mesenchymal stromal cells (tMSCs) are a key cell population to differentiate into adipocyte, which was driven by MRAP and aggravated by aging. We have further uncovered that thymosin-α1 acts as a key upstream factor to induce MRAP expression and adipogenesis in tMSCs in a FoxO1 dependent manner. Importantly, we have confirmed that thymosin-α-FoxO1-MRAP-axis is also required in the adipogenesis by human thymic MSCs.

The thymus is constituted by a myriad of cell types, such as thymocytes, B cells, macrophages, dendritic cells, epithelial cells, fibroblasts and mesenchymal stromal cells^32,33^. Here we, by series of experimental approaches of enzymatic digestion, purification, cultures and passages in the thymus tissues, have isolated and characterized thymic mesenchymal stromal cells with markers of CD31-CD45^−^CD34^−^Aire^−^Ly51^−^EpCAM^−^MHCII^−^CD86^−^CD40^−^SSEA4^−^CD146^–^CD105^+^Sca1^+^CD29^+^. This excludes hematopoietic linage cells (CD45^−^CD34^−^), thymic epithelial cells (Aire^−^Ly51^−^EpCAM^−^), endothelial cells (CD146^−^) and fibroblast (CD31^-^Ly51^-^). We termed these thymus-derived stromal cells as “thymic MSCs (tMSCs)”, which is consistent with other reports^18,20,32^. Despite the similarity of major makers to bone marrow and umbilical cord derived MSCs, tMSCs express few (if any) CD73^+^ cells from mouse. Currently, increasing stem cell researchers suggest that the minimum criteria for defining MSCs established earlier maybe unduly, since the characteristic of MSCs may vary according to the source of tissue^34^. Moreover, the cells identified have been cultured and passaged in vitro for a few days, which may affect the intrinsic marker. So more advanced molecular tools including assessments of the cell transcriptome, proteome, and secretome should be evaluated in creating new definition, especially in single-cell level. We focus on this cell population on thymic adipogenesis since we find that tMSCs are intrinsically more prone to differentiate into adipocytes, providing rational to believe their involvement in the progress of age-related thymic involution.

Based on the functional difference between tMSCs and dpMSCs, we have further identified melanocortin-2 receptor accessory protein (MRAP) as a key factor in mediating adipocyte differentiation by tMSCs. MRAP was originally identified to be a fat-specific protein and highly enriched in differentiated adipocytes^35^. A recent study showed that MRAP is a direct target gene of *PPARγ*, a master transcription factor for adipogenesis^36^. However, we found that reduction of MRAP by gene knockdown or mutant causes profound decrease in *PPARγ* expression in tMSCs during adipogenesis, indicating a positive feedback regulation between MRAP and *PPARγ*. It would be ideal to use Cre-Floxed mice to identify the relationship between MRAP and tMSCs adipogenesis. However, since there is lack of specific marker for tMSCs and thus no specific-cre for tMSCs could be used. We thus chose an alternative approach by generating the systemic MRAP knockout (*Mrap-*KO) mice.

In addition, whether and how MRAP is involved in adipocyte metabolism and/or the lipolytic activation also remain to be determined. Besides adipogenesis, MRAP has been suggested as regulator of receptor trafficking, signaling and ligand specificity^37^. In adrenal cells, MRAP is involved in ACTH-induced activation of the cAMP/PKA pathway by melanocortin 2 receptor (MC2R), leading to glucocorticoid production and secretion^25,38,39^. However, our data demonstrated that the levels of glucocorticoid show no significant change in the thymus of *Mrap*^-/-^ mice compared with age-matched wildtype mice. This suggests that the impaired production of glucocorticoid is not the major factor contributing to the enhanced thymic size in *Mrap*^-/-^ mice. It remains to be determined however whether there is any crosstalk between adipogenesis and glucocorticoid production mediated by MRAP.

Thymosin-α1 is a key upstream factor driving MRAP expression and consequent adipogenesis by thymic MSCs. Thymosin-α1 is chemically synthesized and used in diseases where the immune system is dysfunction. Consistent with our findings, it was reported before that the serum levels of thymosin-α1 are not reduced by thymic involution^40^. Instead, we found that thymosin-α1 increases in the thymus with age, suggesting its role in affecting the process of adipose differentiation by tMSCs during age related involution. The more definite prove of thymosin-α1 role in adipogenesis came from its upregulation of MRAP expression and action through MRAP in tMSCs, as thymosin-α1 fails to increase the adipogenic related differentiation genes and to promote adipogenesis in the *Mrap*^–/–^ tMSCs. The data obtained from injecting thymosin-α1 into WT mice and then examining their thymic adipogenesis compared to untreated WT mice also support that the thymosin-α1 enhances thymic adipogenesis and thymic involution in WT mice (Supplementary Fig. 8).

In understanding the molecular pathway mediating thymosin-α1 signaling to MRAP expression and adipogenesis, we have revealed that the mammalian Fork head box O-1 (FoxO1) plays a key role. Evidence supporting this conclusion include that thymosin-α1 activates p-AKT and increases FoxO1 protein. Importantly, inhibition of FoxO1 blocks MRAP expression and consequent adipogenesis by tMSCs. Our findings are in line of the known feature of FoxO1 in adipocyte differentiation. In this regard, FoxO1 is highly expressed in the adipocytes, where it organizes the transcription of genes related to adipocyte differentiation and trans-differentiation, oxidative stress defense, and lipid metabolism^41^. Notably, several studies have also shown the multifaceted function of FoxO1 in adipocytes, highlighting its signaling role able to maintain energy and redox homeostasis during adipocyte differentiation and nutrient fluctuations^42,43^. Although more work is needed to provide mechanistic insights governing the reciprocal regulation between MRAP and FoxO1 in tMSCs and the possible significance in vivo, we have uncovered a previously unrecognized thymosin-α1-FoxO1-MRAP pathway in driving adipogenesis by tMSCs. Most significantly, we reproduced the function of this pathway in human thymic MSCs, proving a start point to better understand the age-related thymic involution in human.

The current study still has some limitations. It is important to show the change of tMSCs and adipogenesis in older MRAP knockout mice, like older than 18 months of age. However, we observed the abnormally low yield of the *Mrap*^*-/-*^ mice, which is attributed to the increased lethality, including embryonic and neonatal lethality. The generation rate of the *Mrap*^*-/-*^ mice in our study is 3.36% for 3.5 years of breeding. MRAP is highly expressed in the brain and adrenal gland and has been demonstrated to affect the differentiation of progenitor cells within the adrenal gland^44^. Early reports showed that *Mrap*^*-/-*^ mice do not survive after birth without the intervention of in utero corticosterone and had impaired adrenal gland development^25,45^. In addition, most of *Mrap*^*-/-*^ pups died due to impaired maturation of the lungs, which can be rescued by corticosterone administration, indicating that this phenotype is due to corticosterone depletion^25^. We also determined serum and thymic corticosterone levels in our *Mrap*^*-/-*^ mice and found a decrease of corticosterone in serum but not in the thymus (Supplementary Fig. 5). The presence of elevated serum ACTH paired with lower levels of serum corticosterone is suggestive of partial systemic glucocorticoid deficiency. Having been faced with the limitation of the viable *Mrap*^*-/-*^ mice and believed that the KO mice could offer us a unique model to assess thymic adipogenesis mediated by this molecule, we plan to seek the possibility to generate conditional knockout mice in which *Mrap* is deleted only in MSCs in the future.

Significantly, a single cell RNA-sequencing analysis of young and aged human thymus was done and demonstrated an increased number of MSCs and adipocytes with aging (Fig. 8 and Supplementary Fig. 9). Notably, thymic MSCs exhibited a robust potential for adipogenic differentiation. Although previous studies have suggested that adipogenesis in the thymus is caused by a lack of signals, such as epithelial growth factors^46^, our analysis of differentially expressed genes (DEGs) revealed a decrease in some EGF-related gene expression, but increase in other EGF-related genes in both MSCs and adipocytes with age (Supplementary Fig. 9D). Thus, the association between the changes in EGFs and the adipocyte deposition would be worth of further investigation in the future. The differentiation of adipocyte begins with changes in extracellular matrix (ECM) components and cytoskeleton^47^. Single sample gene set enrichment analysis (ssGSEA) revealed that MSCs in old group downregulated ECM organization and collagen formation while upregulated actin cytoskeleton reorganization, fatty acid homeostasis and fat cell differentiation (Supplementary Fig. 9E). Similarly, we noticed the increase of fat cell differentiation and proliferation, fatty metabolism, and lipid storage in adipocytes in old thymuses, and the enrichment of the signaling pathways related to stem cell maintenance and HIPPO signaling in adipocyte of young thymuses (Supplementary Fig. 9E). The interaction analyses indicate that the ECM and adhesion related interaction between MSC and other stromal cells reduced during aging (FN1-ITGA5, FBN1-ITGB1, FBLN1-ITGB1, COL1A1-ITGA11, VCAN-ITGB1) (Supplementary Fig. 9F). Interestingly, PLAU-PLAUR that is upregulated in old group MSC interaction was reported to contribute to plasmin synthesis^48^. In addition, interactions associated with inflammation (IL1B-IL1R1, IL6-IL6ST and PLA2G2A-ITGB1) were enhanced during aging. Moreover, HAS2-CD44 that is upregulated during aging was reported to be associated with adipocyte differentiation^49^. However, the potential roles and mechanisms of these interactions and associations in MSC adipogenesis warrant further investigation in the future. Nevertheless, these additional and exciting single-cell RNA data on human thymus further validate our findings that thymic MSCs play an important role in the adipogenesis during thymic involution.

## Methods

### Animals and reagents

All mice used in the study were housed under the specific pathogen-free conditions in the animal facilities of the Model Animal Research Center (MARC) of Nanjing University (Nanjing, China), or the animal facility in National Institute of Dental and Craniofacial Research (NIDCR). All procedure of animal studies was performed under the Animal Research Committee-approved protocols by the MARC of Nanjing University and under the National Institutes of Health (NIH) guidelines for use and care of live animals and were approved by the Animal Care & Use Committee (ACUC) of the NIDCR. C57BL/6 (B6) female mice were purchased from MARC of Nanjing University, or from Jackson Laboratory. *Mrap*^*-/-*^ mice were created by CRISPR-Cas9 mediated genome engineering. We designed the sgRNA sequence for the first exon of *Mrap* gene, selected its target site as CAGGGGGCCGTTCCCCTGTA, constructed the Cas9/sgRNA plasmid, and microinjected it into the fertilized eggs of C57BL/6 mice. We selected F0 generation *Mrap*^*-/-*^ mice to mate with WT mice for breeding and subsequent experiments. Mouse DNA was extracted from the tail with a length of 0.5 cm, and 2 μL DNA extraction was detected for DNA purity and concentration, then was used for PCR amplification. The *Mrap* genome DNA sequence was found in NCBI. The upstream and downstream primers were designed according to the corresponding primer design principles and submitted to GemPharmatech Co., Ltd. for synthesis. The upstream primer sequence was 5′- CATGCACATCGCAGAGTACATACG-3′, the downstream primer sequence was 5′-GCACGGCATTGTGTTCTACAGC-3′, and the product length was 650 bp. PCR reaction adopts 25 μL system, containing 12.5 μL 2 × Taq Master Mix, 1 primer for upstream and 1 primer for downstream (10 pmol/μL), DNA template 100 ng/μl, supplemented with water without RNA enzyme 25 μL system. PCR amplification was performed using the following procedure: pre denaturation at 95 °C for 5 min; Then enter the cycle at 98 °C for 30 s, 65 °C for 30 s, 72 °C for 45 s, and cycle for 40 times. Fully extend at 72 °C for 5 min. Furthermore, the acquisition rate (Supplementary Table 2), survival rate, and the phenotypes of the *Mrap*^*-/-*^ mice have been identified (Supplementary Fig. 2 and 3).

To evaluate the role of thymic environment factors in thymus involution, recombinant thymosin-α1 (Abcam, ab42247), ACTH (1-39) (R&D, #3492), and FoxO1 inhibitor (MCE, #AS1842856) were used in vivo or in vitro as indicated.

### Isolation and culture of MSCs

For human thymus, with the consent of the patients, fresh thymus tissue was collected as surgical waste from undergoing cardiac surgery according to the Ethics Committee of Affiliated Drum Tower Hospital of Nanjing University Medical School (2021-453) and the Ethics Committee of the West China Hospital (2020-39), Sichuan University, which were collected and transferred to RPMI1640 medium supplied with 10% FBS on ice, the tissues were cut into small pieces to release thymocytes. After thymocytes were collected, the remaining tissues were washed with RPMI1640 for 2–3 times and subjected to digestion with pre-warmed RPMI1640 containing 0.1 g/mL collagenase and 100 ug/mL DNase at 37 °C for 30–40 min. The cell suspension was passed through 70 μm cell strainer and combined with thymocytes collected previously. The cells were centrifuged at 500 g for 6 min and suspended with RPMI1640 medium for cell counting, staining and sorting. For purification and culture of MSCs, the cells were plated on 60-mm culture dishes and allowed to proliferate in DF12 medium, supplemented with 20% FBS plus 100 U penicillin/streptomycin (Sigma) and cultured at 37 °C in a 5% CO_2_ humidified incubator. After 2 days of culture, nonadherent cells were removed, and adherent cells were washed and incubated until confluence in DF12 medium. Medium was then changed every 3 days and serially expanded.

For dental pump MSCs isolation, we firstly extracted the lower incisors from mandibles of the mouse under stereomicroscope. Then we removed the dentin from the apical roots to expose the dental pulps. Next, we pull out the dental pulps very carefully and put them into a tube with c-DMEM on ice for future use. Dental pulp tissues were digested and cultured as thymus tissues as above.

### CFSE dilution assay

For carboxy-fluorescein diacetate succinimidyl ester (CFSE)-labeling assay, 10^6^ cells/mL mouse spleen derived monocytes were incubated with 3 μM CFSE in phosphate-buffered saline (PBS)/0.5% bovine serum albumin (BSA) at 37 °C for 15 min. Cells were washed three times with fresh pre-cold complete 1640 medium and resuspended in complete 1640 for further culture. After 5 to 6 days culture with MSCs or alone, cells were harvested for examining the CFSE negative cells by flow cytometry.

### Differentiation analysis

The differentiation of MSCs were performed by using induction media, either MesenCult™ Osteogenic Differentiation Kit (Stem cell Technologies, #05504 for mouse or #05465 for human) or MesenCult™ Adipogenic Differentiation Kit (Stem cell Technologies, #05507 for mouse or #05412 for human) that were applied to cell populated tMSCs or dpMSCs directly. Cells were cultivated in induction at the confluence of 80%. For the adipogenic differentiation tests, Oil Red O (Sigma) staining was performed to verify adipogenic differentiation, samples were fixed in 60% isopropanol for 5 min and then a solution of Oil Red O and water (2:3) was placed in contact with the samples for additional 5 min. Images were collected with an upright optical microscope (Olympus) in brightfield. For osteoblastic differentiation, MSCs were cultured under osteogenic culture condition, containing 2 mM β-glycerophosphate (Sigma-Aldrich), 100 μM L-ascorbic acid 2-phosphate (Wako), and 10 nM dexamethasone (Sigma-Aldrich) in the growth medium. After 2 weeks induction, ALP staining (Sigma-Aldrich) was performed to detect matrix mineralization, and the stained areas were quantified by NIH ImageJ software and shown as a percentage of the total area.

### RNA isolation and sequencing

Total RNA was extracted using the RNeasy mini kits (QIAGEN), purified using Direct-zol RNA MiniPrep kit (Zymo Research). Next-generation libraries were prepared using the VAHTS^TM^ mRNA-seq V2 Library Prep Kit for Illumina (Vazyme, #NR601). RNA-seq libraries were run on an Illumina HiSeq X-Ten next-generation sequencer. Analysis of RNA-seq data was done using the DESeq package in R.

### Real-time (RT)-qPCR

Total RNA was isolated by using RNA isolater (Vazyme, R401-01-AA) following the instructions according to the manufacturers. RT-qPCR reactions were conducted using SYBR^®^ Green Master Mix (Low ROX Premixed) (Vazyme, #Q131) on the Q6 Real-time PCR System (Applied Biosystems, United States) according to the instruction. Relative expression levels of the indicated genes were calculated by using the 2^−△△Ct^ method following normalization to GAPDH. Primers for RT-qPCR in this study see (Supplementary Table 3).

### Small interfering RNA (siRNA) synthesis and transfection

The expression of MRAP by tMSCs derived from human or mouse were downregulated by RNA interference technique. siRNA specifically targeting MRAP and control siRNA (scrambled) were designed and synthesized by GenePharma (Suzhou, China). The human MRAP siRNA sequences were GCCCACAAACAUUCCAUCGTT-CGAUGGAAUGUUUGUGGGCTT, human GAPDH siRNA sequences were CACUCAAGAUUGUCAGCAATT-UUGCUGACAAUCUUGAGUGAG, mouse MRAP siRNA were GCUCACCAGCUAUGAGUAUTT-AUACUCAUAGCUGGUGAGCTT, mouse GAPDH siRNA were UGACCUCAACUACAUGGUUTT-AACCAUGUAGUUGAGGUCATT, negative control sequence: UUCUCCGAACGUGUCACGUTT-ACGUGACACGUUCGGAGAATT. The siRNAs were transfected into cells with Lipofectamine 2000 (Invitrogen) according to the instruction and the interference effect was examined by RT-qPCR.

### ELISA assay

Thymic environment factors of serum, urine and thymus of mice were measured using a mouse enzyme-linked immunoassay (ELISA) method. ELISA Kit of ACTH (ab263880) and insulin (ab277390) were purchased from Abcam, mouse CORT (Corticosterone) ELISA Kit (E-OSEL-M0001) was purchased from Elabscience Biotechnology (China). For detection of thymosin-α1 levels, the water-soluble protein extracts of serum and urine as well as thymus with coating buffer were inoculated in a 96-well ELISA plate at 4^o^C overnight, the mixtures were transferred and the wells were incubated with blocking buffer for 1 h at 37 °C. After washing with PBST, levels of thymosin-α1 of mice were detected by adding rabbit anti-thymosin-α1 antibody (Bioss Biotechnology, China; bs-0226R; 1:2000) and goat anti-rabbit IgG antibody (Bioss Biotechnology; bs-0295G; 1:2000). The synthetic thymosin-α1 was used as standard. The absorbance was read at 450 nm with a reference of 630 nm with Microplate Reader.

### Western blot analysis

Total proteins were extracted from cells using radioimmunoprecipitation assay (RIPA) lysis buffer (Beyotime Institute of Biotechnology, China; #P0013C) plus 1% Protease Inhibitor Cocktail (APExBIO, Houston, TX, USA; #K1007), and protein concentrations were measured by the bicinchoninic acid (BCA) assay. Equal amounts of proteins were denatured at 100^◦^C, separated by sodium dodecyl sulfate polyacrylamide gel electrophoresis (SDS-PAGE), and transferred onto polyvinylidene fluoride (PVDF) membranes (Millipore, United States). Afterward, the membranes were blocked with 5% (w/v) skim milk at room temperature and immunoblotted overnight at 4 ^◦^C with rabbit polyclonal antibody against MRAP (Proteintech, #19512-1-AP), FABP4 (CST, #2120), pFoxO1 (CST, #9461), AKT (CST, #4691), p-AKT (CST, #13038), and mouse monoclonal antibody against FoxO1 (CST, #14952), α-tubulin (Proteintech, #66031-1-Ig) and GAPDH (Proteintech, #60004-1-Ig) according to the instruction, respectively. Then, appropriate horseradish peroxidase (HRP)-conjugated secondary antibodies were applied, and the protein bands were detected by the enhanced chemiluminescence (ECL) detection system.

### Flow cytometry analysis

For cell surface markers of tMSCs analysis, the detached cells were washed and resuspended in phosphate buffered saline (PBS) after trypsinization. ~10^6^ cells were incubated with antibodies against Aire-Alexa Fluor 488, Ly51-FITC, EpCAM-PE, MHCⅡ-PE, CD86-PE, CD40-PE, CD73-PE, CD29-FITC, CD105-APC, Sca1-FITC, CD31-FITC, CD34-PE, CD45-PE, CD146-PE and SSEA4-PE. For phenotype identification of *Mrap*^*-/-*^ mice, thymocytes were phenotyped by flow cytometry with live/dead-eF780 and mAbs against mouse CD4-BV711, CD5-APC, CD8a-Percp Cy5.5, CD25-APC, CD44-BV421, CD45-BV510, CD45R-Alexa Fluor 700, and CD69-BV605, CD105-PE, Sca1-FITC or their isotype-control antibodies. Only singlets and live/dead-eF780 negative cells were included in the analysis. All surface antibody incubations were performed at 4 °C for 30 min. Cells were washed with PBS containing 0.1% BSA after incubation. The Aire intracellular staining was performed according to manufacturer’s instructions. Fresh cells were fixed and permeabilized with Fixation/Permeabilization Solution in the dark at 4 °C for 30 min. After washing twice with permeabilization buffer, the cells were incubated with anti-Aire Ab for 30 min at 4 °C. Finally, the cells were assayed in a FACS flow cytometer (BD Fortessa).

### Single cell library construction

For scRNA-seq, fresh cells isolated from human thymus were sorted as 7-AAD^-^CD235a^-^CD45^+^ and 7-AAD^-^CD235a^-^CD45^-^ using Aria III (BD). The sorted CD45^+^ and CD45^-^ cells were combined with the ratio at 6:4 for 10× single cell RNA-sequencing and 5’-TCR repertoire sequencing. Number and viability of the sorted thymic cells were measured. For 10× single cell RNA-sequencing and 5’-TCR repertoire sequencing, 20,000 live sorted thymic cells were loaded on to each of the Chromium Controller (10× Genomics). Single cell cDNA and unique molecular identifier (UMI) tagged 5’ scRNA-Seq libraries and 5’-TCR repertoire Seq libraries were prepared using 10× Genomics platform.

### Upstream analysis of scRNA-Seq data

Sequencing data from 10× Genomics were demultiplexed and mapped against the GRCh38 genome and quantified using the *CellRanger* software with default settings to generate raw gene expression matrix. Cells with fewer than 200 genes detected and more than 10% mitochondrial reads were considered as low qualities cells and were filtered out. Doublets identified by doublet detection were also removed^50^. Genes that were expressed in fewer than 3 cells were removed as well. After basic quality control, contaminated cells that expressed genes of multi-lineage and low-quality cells with relative low total counts but high mitochondrial reads were removed based on clustering analysis.

### Cell heterogeneity detection and annotation

The Seurat package^51^ was used to process the raw count data, reduce dimensionality, cluster, and perform differential expression with accustomed protocol unless mentioned specially. Dimensionality of the data was reduced by principal component analysis (PCA) first, then we generate harmony embedding with batch effect corrected^52^, which was applied to downstream analysis. We manually annotate cell type based on genes that were differentially expressed across different cell types with the Find Markers function (Wilcoxon rank sum test, *p* values adjusted for multiple testing using the Bonferroni correction), genes with corrected *p* value < 0.01 and fold change > 1.5 were consider to be differentially expressed.

### Aging associated genes and pathways analysis

Wilcoxon rank-sum test and single sample gene set enrichment analysis (ssGSEA)^53^ were applied to detect aging associated genes and pathways, respectively. Pathway database utilized in ssGSEA was obtained from MSigDB. Wilcoxon rank sum test was used to detect the significant differences, *p* value was adjusted for multiple testing using the Benjamini & Hochberg correction. Genes with *p* value < 0.05 and pathways with corrected *p* value < 0.05 were consider to be differentially changed during aging.

### Crosstalk analysis

To examine cell-cell communication that was significantly changed with aging from scRNA-seq data, we combined the previously published ligand-receptor pairs into a new database and used permutation method to test the significance^54–56^. In brief, the interaction score ($S$) of certain ligand-receptor pair in a certain cell-cell group pair was defined as follow:

$$S=\,{\bar{e}}_{i,\,l}\times {\bar{e}}_{j,\,r}$$

Where ${\bar{e}}_{i,\,l}$ and ${\bar{e}}_{j,\,r}$ represents the mean expression of ligand gene $l$ in cell group $i$ (e.g., a stromal cell subset in a certain age group) and the mean expression of receptor gene $r$ in certain cell group $j$ (e.g., another stromal cell subset in the same age group), respectively. Later, we randomly permuted the age group label within each cell subset for N times (N was set to be 1000 by default) and calculated the corresponding interaction score ${S}_{p}$. *p* value of a certain interaction was defined as follow:

$$p=\frac{n}{N}$$

Where $n$ represents the counts of ${S}_{p}$ that were as or higher than $S$. Interactions with the *p* value less than or equal to 0.01 were considered to be significantly up regulated. Cell-cell communications along with trajectory of adipocyte differentiation were inferred in a similar way.

### Trajectory analysis

The raw count matrix containing MSC, adipocyte 1 and adipocyte 2 were imported into Monocle 3^57^. UMAP dimensional reduction result was extracted from the Seurat pipeline. Cells were clustered with cluster cells using ‘Leiden’ algorithm with the nearest neighbors set to 20 and resolution set to 4e-4. The trajectory graph was learned on the Monocle-derived clusters by calling learn graph. Cells on the UMAP plot are colored by Seurat-derived clusters. Pseudo time was determined using MSC as the starting point.

### Histopathology & immunofluorescence analysis

For histological analysis, tissues were fixed using 10% buffered formalin. After fixation, the tissues were dehydrated in ethanol, embedded in paraffin, and stained with Hematoxylin and eosin (H&E). All slides were marked and then interpreted thoroughly for information. For cellular immunofluorescence staining, cells were fixed with 4% paraformaldehyde (PFA) in PBS for 20–30 min and permeabilized in 0.25% Triton X-100 (Sigma) for 10 min. Nonspecific binding sites were blocked with 10% goat serum for 30 min at room temperature. Cells were then incubated overnight at 4 °C with primary antibody (Anti-FoxO1 antibody, dilution 1:100). After three rinses in PBS, cells were exposed to goat anti-mouse immunoglobulin G conjugated to FITC (dilution 1:1000) for 1 h at room temperature, followed by nuclear staining with DAPI for 10 min. After three rinses in PBS, coverslips were mounted on slides. The cells on coverslips were examined by confocal laser scanning microscopy using an Olympus FV3000 Microscope.

For multiplex immunofluorescence (mIF) examination of tMSCs distribution in thymus tissue sections, titration of fluorophore-conjugated tyramide signal amplification (TSA) buffer (Servicebio, China) was performed using a modified IHC protocol^58^. In brief, deparaffinised 5 mm thymus sections were subjected to laboratory microwave before incubation with hydrogen peroxide to quench endogenous peroxidase activity that would otherwise interfere with the HRP-tyramide activation step. Antibody-based marker detection was then conducted sequentially according to the labeling order of EpCAM (GB11274,1:3000)/CD105 (GB113503, 1:3000)/Sca1 (GB11988, 1:2000)/CD90 (GB113753, 1:200). Briefly, slides were incubated with a single primary antibody in antibody diluent containing background-reducing components followed by application of a polymeric HRP-conjugated secondary antibody. The appropriate Opal fluorophore-conjugated TSA (FITC-TSA/G1222, CY3-TSA/G1223, 647-TSA/G1224) was then added at 1:2000 dilution. Slides were rinsed with washing buffer after each step. Following the heat-stable deposition of the TSA-conjugated fluorophore around the marker of interest, slides were again subjected to heat-induced epitope retrieval to strip the primary and secondary antibodies bound to the tissue, ready for labeling of the next marker. These steps were repeated until all four markers were labeled; finally, DAPI was added as a nuclear counterstain. Slides were mounted in anti-fluorescence quenching sealed buffer (Servicebio,G1401) and cured in the dark at room temperature for 24 h prior to storing at 4 °C. Fluorescence images were acquired using a Panoramic MIDI digital slice scanner (3DHistech), and then analyzed by Caseviewer 2.4 software.

### Statistical analysis

Pairwise differences were measured using two-tailed independent student’s t-tests. If the data did not meet this test, a Mann-Whitney U- test was used. Statistical significance between groups of 3 or more was determined by a one-way ANOVA, followed by the Tukey’s multiple comparison test. Data are presented as the mean ± SD. Statical correlation was measured using the Pearson correlation coefficient (two-tailed, confidence interval (CI) = 95%). Statistical analysis was performed using GraphPad Prism 9. Values of *p* < 0.05 were considered as significantly different.

### Reporting summary

Further information on research design is available in the Nature Portfolio Reporting Summary linked to this article.

### Reporting summary

Further information on research design is available in the Nature Portfolio Reporting Summary linked to this article.

## Supplementary information

Supplementary Information

Reporting Summary

Peer Review File

## Data Availability

The authors declare that the data supporting the findings of this study are presented within the paper and its Supplementary Information files. Source data are provided with this paper. Data for bulk RNA-seq (GSE183216) has been uploaded on public database and can be found by this link: (https://www.ncbi.nlm.nih.gov/geo/query/acc.cgi?acc=GSE183261). Data for scRNA-seq (GSE231906) has been uploaded on public database and can be found by this link: (https://www.ncbi.nlm.nih.gov/geo/query/acc.cgi?acc=GSE231906.).
