# Supplementary material for: MRAP mediated adipocyte differentiation by thymic mesenchymal stromal cells contributes to thymic involution
Source: Nat Commun. 2025 Nov 20;16:10210. doi: 10.1038/s41467-025-64973-z (PMC12635226; doi:10.1038/s41467-025-64973-z)
Supplement: Supplementary file 1 — Supplementary Information [file 41467_2025_64973_MOESM1_ESM.pdf]

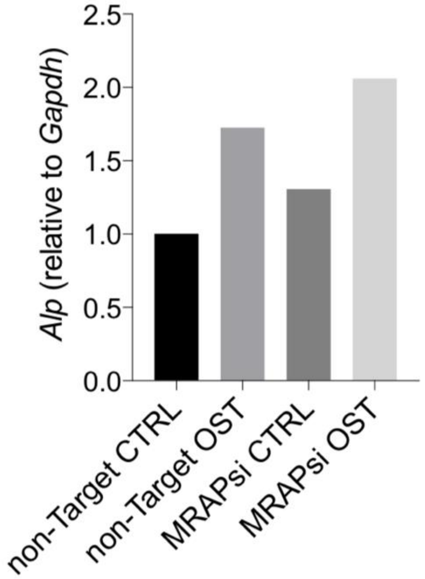

Alp (relative to Gapdh)
2.5
2.0
1.5
1.0
0.5
0.0
non-Target CTRL
non-Target OST
MRAPsi CTRL
MRAPsi OST

Supplementary Fig. 1: MRAP does not mediate osteogenesis in tMSCs

Osteoblastic related genes *Alp* was detected in siMRAP-transfected tMSCs by RT-qPCR.

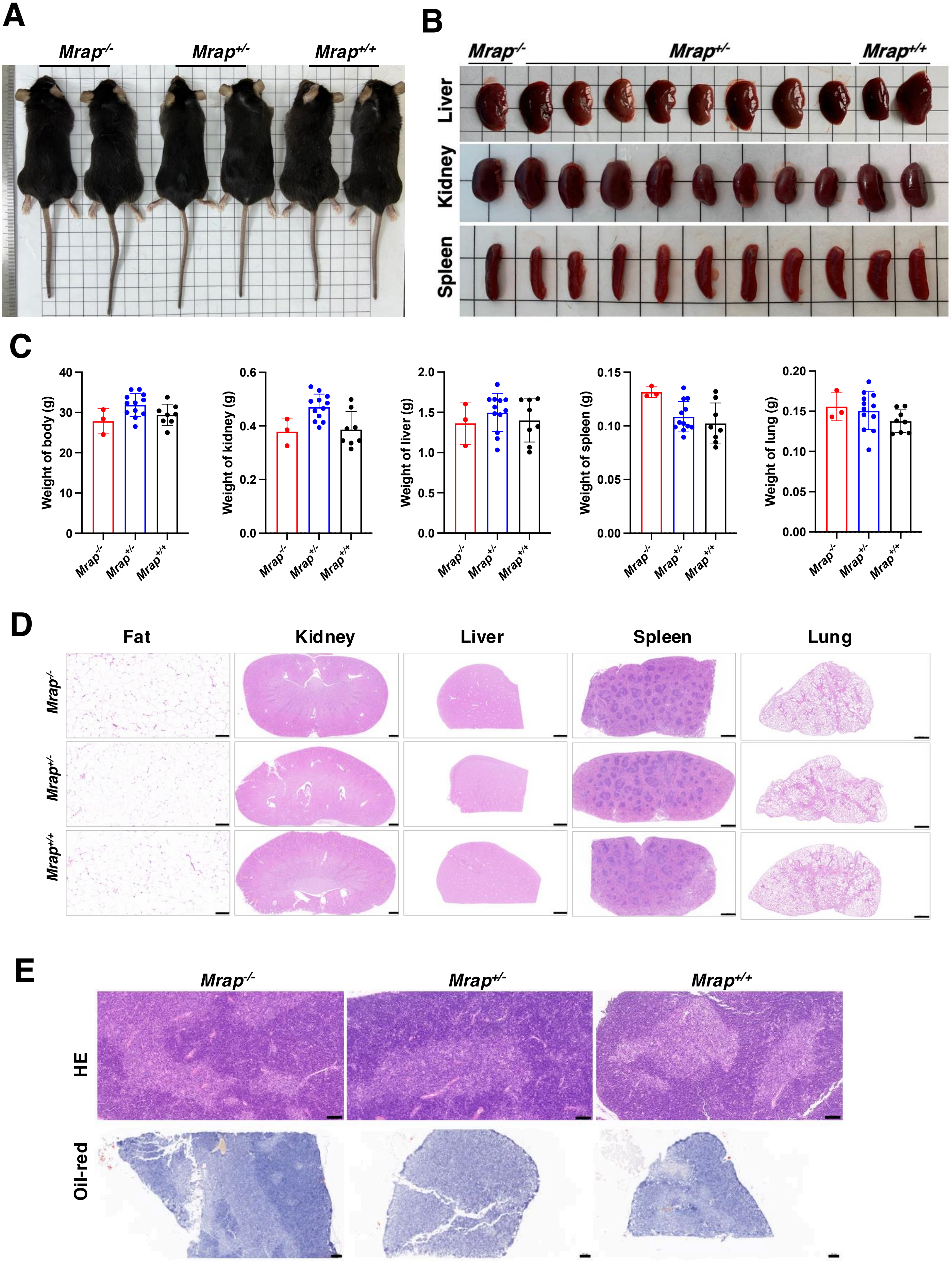

A
Mrap-/-
Mrap+/-
Mrap+/+
B
Liver
Kidney
Spleen
C
Weight of body (g)
Weight of kidney (g)
Weight of liver (g)
Weight of spleen (g)
Weight of lung (g)
D
Fat
Kidney
Liver
Spleen
Lung
E
HE
Oil-red

Supplementary Fig. 2: Phenotype identification and histopathological examination of *Mrap*$^{-/-}$ mice

(A) Representative images of a homozygous (*Mrap*$^{-/-}$, n=3, female) and heterozygous (*Mrap*$^{+/-}$, n=12, female) *Mrap* mutant mouse at the age of 7 months compared to a wild type (*Mrap*$^{+/+}$, n=8, female).

(B) Photograph of liver, kidney and spleen isolated from the *Mrap*$^{-/-}$ mice and *Mrap*$^{+/+}$ mice.

(C) Weights of the body and organs (liver, spleen, kidney, and lung) isolated from *Mrap*$^{-/-}$, *Mrap*$^{+/-}$ and *Mrap*$^{+/+}$ mice.

(D) Representative histology with H&E (hematoxylin and eosin staining) of the organs (fat, kidney, liver, spleen, and lung) from young *Mrap*$^{-/-}$, *Mrap*$^{+/-}$ and wild-type mice (7 weeks old, female). Scale bar, 100 µm for fat, 500 µm for kidney and spleen, 1000 µm for liver and lung.

(E) Representative histology with H&E and oil-red staining of the thymus from young *Mrap*$^{-/-}$, *Mrap*$^{+/-}$ and wild-type mice (7 weeks old). Scale bar, 100 µm.

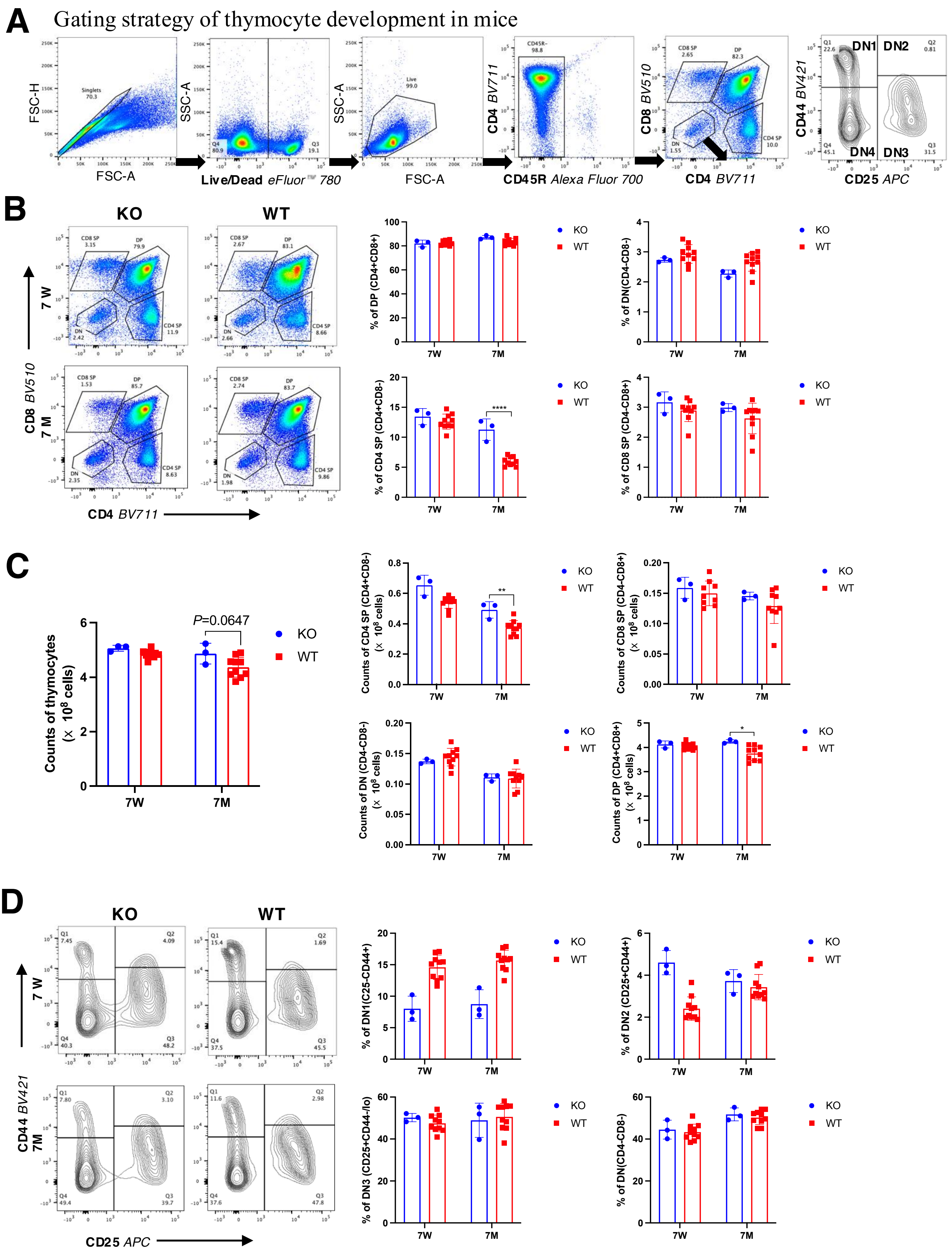
A
Gating strategy of thymocyte development in mice
FSC-H
FSC-A
Singlets 70.3
SSC-A
Live/Dead eFluor™ 780
SSC-A
Live 99.0
FSC-A
CD4 BV711
CD45R- 98.8
CD45R Alexa Fluor 700
CD8 BV510
CD4 BV711
CD8 SP 2.65
DP 82.3
DN 1.55
CD4 SP 10.0
CD44 BV421
CD25 APC
DN1
DN2
DN3
DN4
B
KO
WT
7 W
7 M
CD8 BV510
CD4 BV711
% of DP (CD4+CD8+)
% of DN(CD4-CD8-)
% of CD4 SP (CD4+CD8-)
% of CD8 SP (CD4-CD8+)
7W
7M
C
Counts of thymocytes (× 10^8 cells)
P=0.0647
Counts of CD4 SP (CD4+CD8-) (× 10^8 cells)
Counts of CD8 SP (CD4-CD8+) (× 10^8 cells)
Counts of DN (CD4-CD8-) (× 10^8 cells)
Counts of DP (CD4+CD8+) (× 10^8 cells)
D
KO
WT
7 W
7M
CD44 BV421
CD25 APC
% of DN1(C25-CD44+)
% of DN2 (CD25+CD44+)
% of DN3 (CD25+CD44-/lo)
% of DN(CD4-CD8-)

Supplementary Fig. 3: Effect of *Mrap* deficiency on the thymocytes

(A) Gating strategy of thymocytes development in mice was shown.

(B) The ratios of the major populations of DN, DP, CD4 SP, and CD8 SP cells in thymocytes of *Mrap*$^{-/-}$ and WT mice (7 weeks and 7 months old, female). Thymocytes were measured by flow cytometry with live/dead-eF780 and mAbs against mouse CD4, CD5, CD8, CD25, CD44, CD45R, and CD69. Only singlets and live/dead-eF780 negative cells were included in the analysis. DN, double negative, $CD4^{-}CD8^{-}$; DP, double positive, $CD4^{+}CD8^{+}$; SP, single positive.

(C) Absolute numbers of total thymocytes and the DN ($CD4^{-}CD8^{-}$), DP ($CD4^{+}CD8^{+}$), CD4 SP ($CD4^{+}CD8^{-}$) and CD8 SP ($CD4^{-}CD8^{+}$) thymic subsets in *Mrap*$^{-/-}$ and WT mice between 7-weeks and 7-months of age. DN, double negative; DP, double positive; SP, single positive.

(D) The frequency of DN T cells in early T-cell development. DN1-4 populations are defined by CD44 and CD25 expression, as indicated. n = 3-18 female mice/group pooled from two independent experiments; Two-tailed Student's t test was performed within same age groups. ns, $p > 0.05$; *$p < 0.05$; **$p < 0.01$

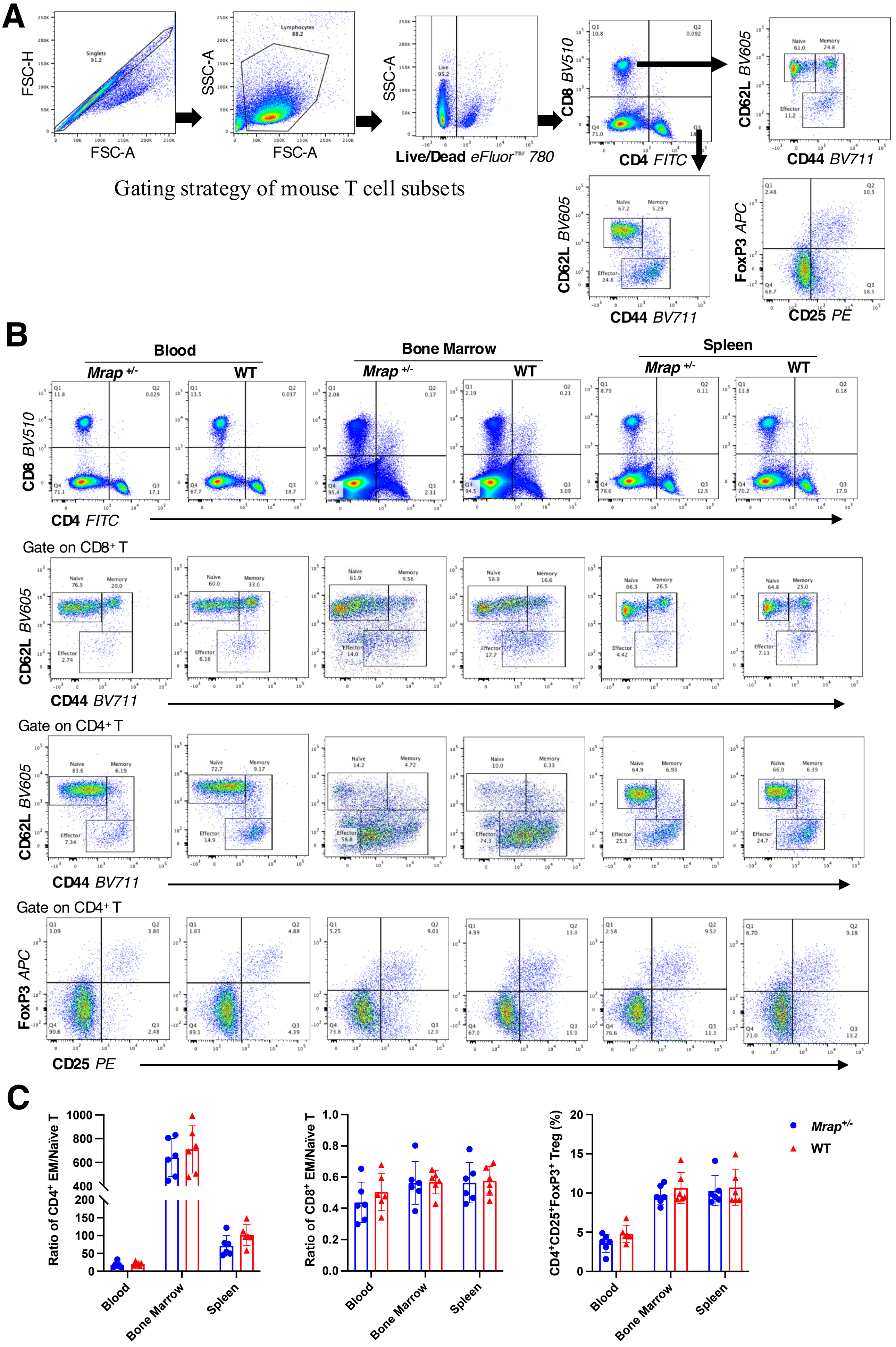
A
FSC-H
FSC-A
Singlets 91.2
SSC-A
Lymphocytes 88.2
Live 95.2
Live/Dead eFluor™ 780
CD8 BV510
CD4 FITC
CD62L BV605
CD44 BV711
FoxP3 APC
CD25 PE
Gating strategy of mouse T cell subsets
B
Blood
Bone Marrow
Spleen
Mrap +/-
WT
Gate on CD8+ T
Gate on CD4+ T
C
Ratio of CD4+ EM/Naïve T
Ratio of CD8+ EM/Naïve T
CD4+CD25+FoxP3+ Treg (%)
Blood
Bone Marrow
Spleen
Mrap+/-
WT

Supplementary Fig. 4：Analysis of T cell subsets changes in *Mrap*$^{+/-}$ mice

(A) Gating strategy of T cell subsets in mice was shown.

(B) Flow cytometry plots of CD4^{+}, CD8^{+}, naïve, memory, and effector T cells and Treg cells in blood, bone marrow and spleen tissues from a representative 10-month-old *Mrap*$^{+/-}$ mice and age-matched WT mice.

(C) Ratios of effector memory (EM)/ Naïve T in CD4^{+} and CD8^{+} T cells and the frequency of Treg cell subsets in blood, bone marrow and spleen tissues in *Mrap*$^{+/-}$ and WT mice.

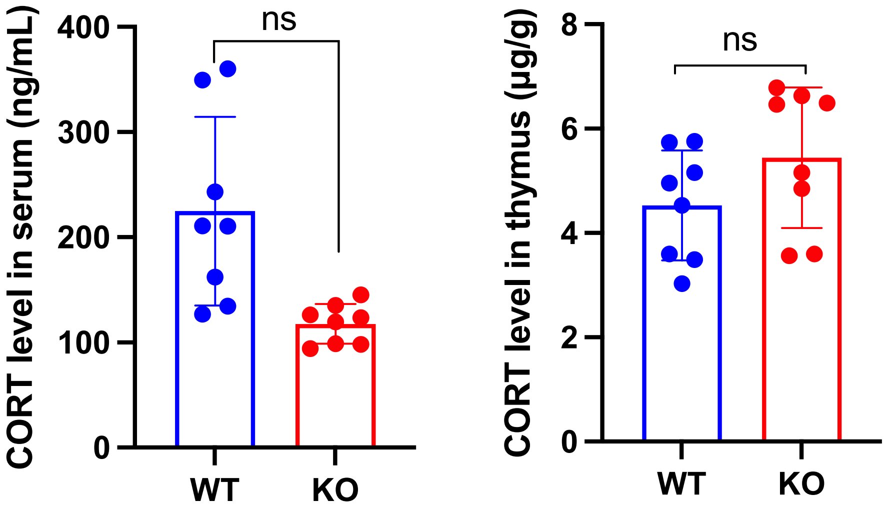

CORT level in serum (ng/mL)
400
300
200
100
0
ns
WT
KO
CORT level in thymus (µg/g)
8
6
4
2
0
ns
WT
KO

Supplementary Fig. 5：The levels of glucocorticoid in the thymus of *Mrap*-KO mice

ELISA assay analysis of serum and intrathymic corticosterone (CORT) levels of WT and *Mrap*$^{-/-}$ mice. Final intrathymic corticosterone levels were represented by the ratio of hormone concentration in supernatants and thymus mass (n= 8 female mice/group).

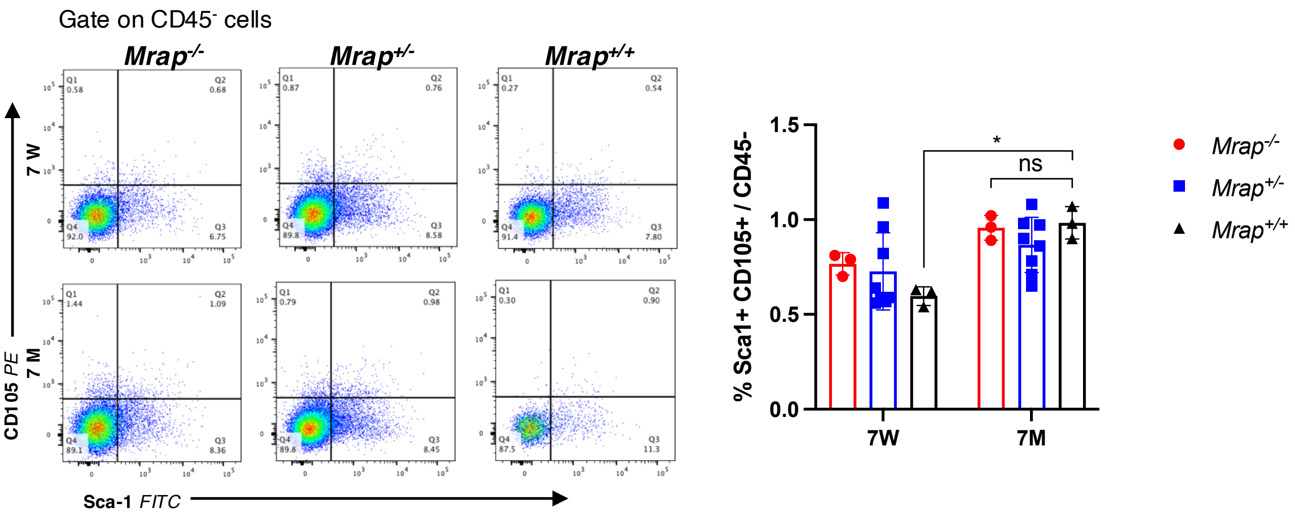

Gate on CD45- cells
Mrap-/-
Mrap+/-
Mrap+/+
7 W
7 M
CD105 PE
Sca-1 FITC
Q1 0.58
Q2 0.68
Q3 6.75
Q4 92.0
Q1 0.87
Q2 0.76
Q3 8.58
Q4 89.8
Q1 0.27
Q2 0.54
Q3 7.80
Q4 91.4
Q1 1.44
Q2 1.09
Q3 8.36
Q4 89.1
Q1 0.79
Q2 0.98
Q3 8.45
Q4 89.8
Q1 0.30
Q2 0.90
Q3 11.3
Q4 87.5
% Sca1+ CD105+ / CD45-
1.5
1.0
0.5
0.0
7W
7M
*
ns
Mrap-/-
Mrap+/-
Mrap+/+

Supplementary Fig. 6: Effect of *Mrap* deficiency to tMSCs composition in the thymus

Representative contour plots showing the percentage of $Sca1^{+}CD105^{+}$ cells among total live CD45 negative thymic stromal cells in $Mrap^{-/-}$, $Mrap^{+/-}$ and $Mrap^{+/+}$ mice with 7 weeks and 7 months old. n = 3-18 female mice/group pooled from two independent experiments; ns, $p > 0.05$; $*p < 0.05$

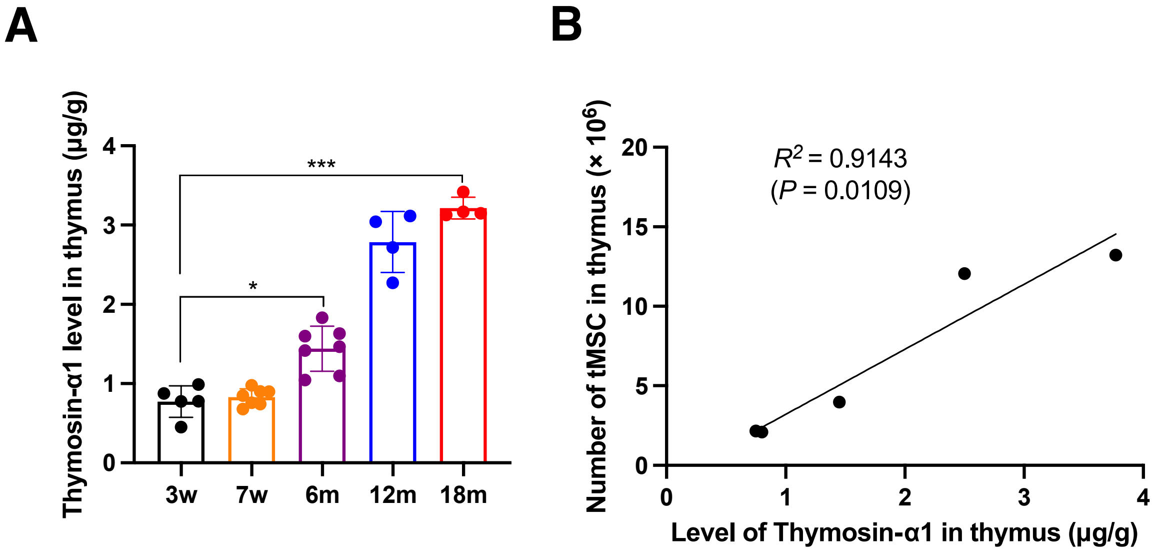
A
B
Thymosin-α1 level in thymus (μg/g)
***
*
3w
7w
6m
12m
18m
Number of tMSC in thymus (× 10^6)
R^2 = 0.9143
(P = 0.0109)
Level of Thymosin-α1 in thymus (μg/g)

Supplementary Fig. 7：The correlation between thymosin-α1 level and the number of tMSCs in mice

(A) The level of thymosin-α1 in the thymus tissue of mice of different ages. (The result is representative of three independent experiments, n = 4-7 female mice per age group/experiment).

(B) The correlation between thymosin-α1 level and the number of tMSCs in WT mice at different ages.

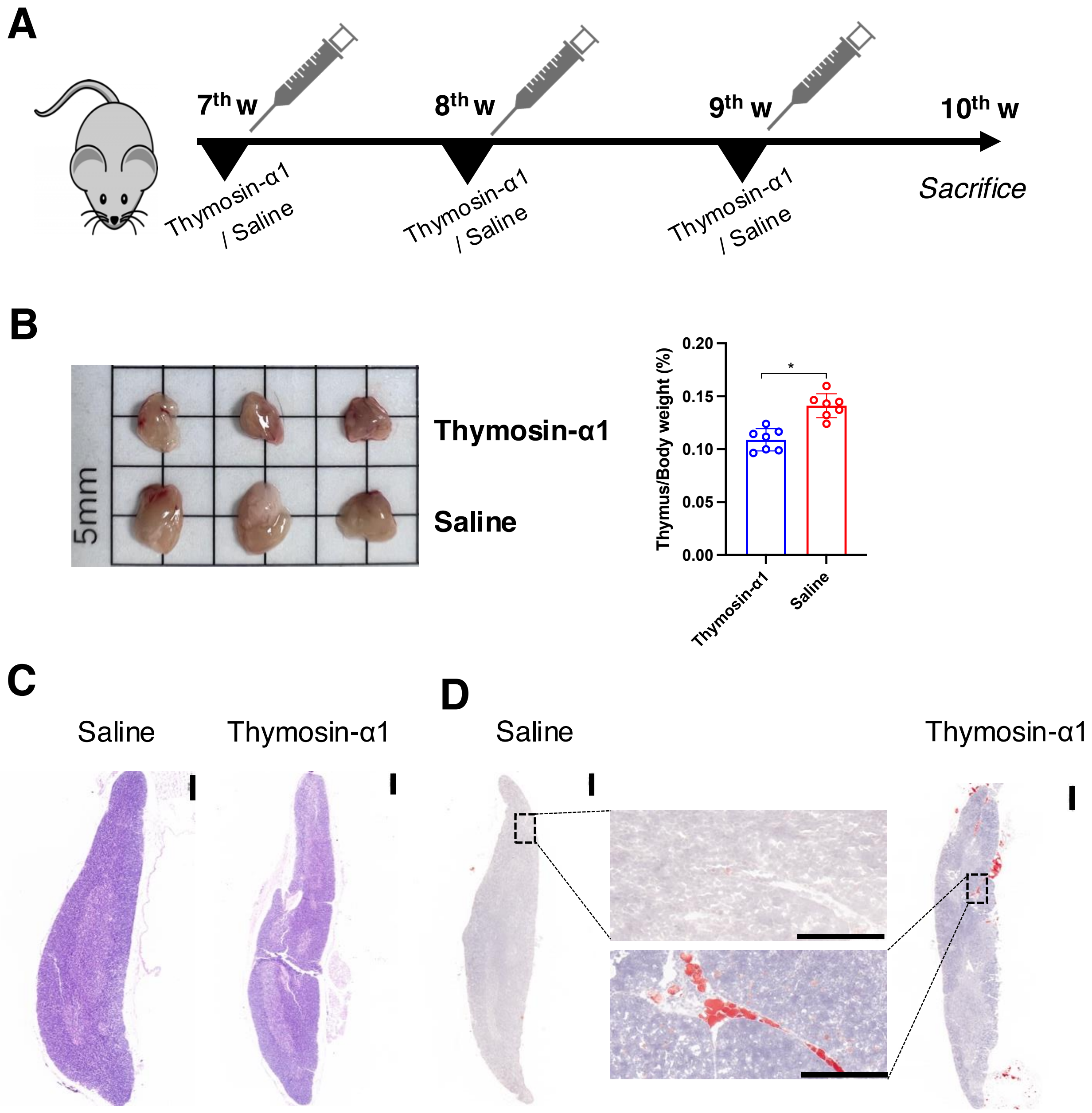
A
7th w
8th w
9th w
10th w
Thymosin-α1 / Saline
Thymosin-α1 / Saline
Thymosin-α1 / Saline
Sacrifice
B
5mm
Thymosin-α1
Saline
Thymus/Body weight (%)
0.20
0.15
0.10
0.05
0.00
*
Thymosin-α1
Saline
C
Saline
Thymosin-α1
D
Saline
Thymosin-α1

Supplementary Fig. 8：The contribution of thymosin-α1 to adipogenesis in the thymus

(A) Overview of the repeated injections performed in 7-week-old B6 female mice (n = 7 female mice per group).

(B) Representative photographs of thymus organs in mice with thymosin-α1 and saline administration were shown. The ratio of thymus/body weight were summarized in each group.

(C, D) Representative histopathology with H&E **(C)** and oil-red staining **(D)** of the thymus from mice with thymosin-α1 and saline administration. Scale bar, 200 μm.

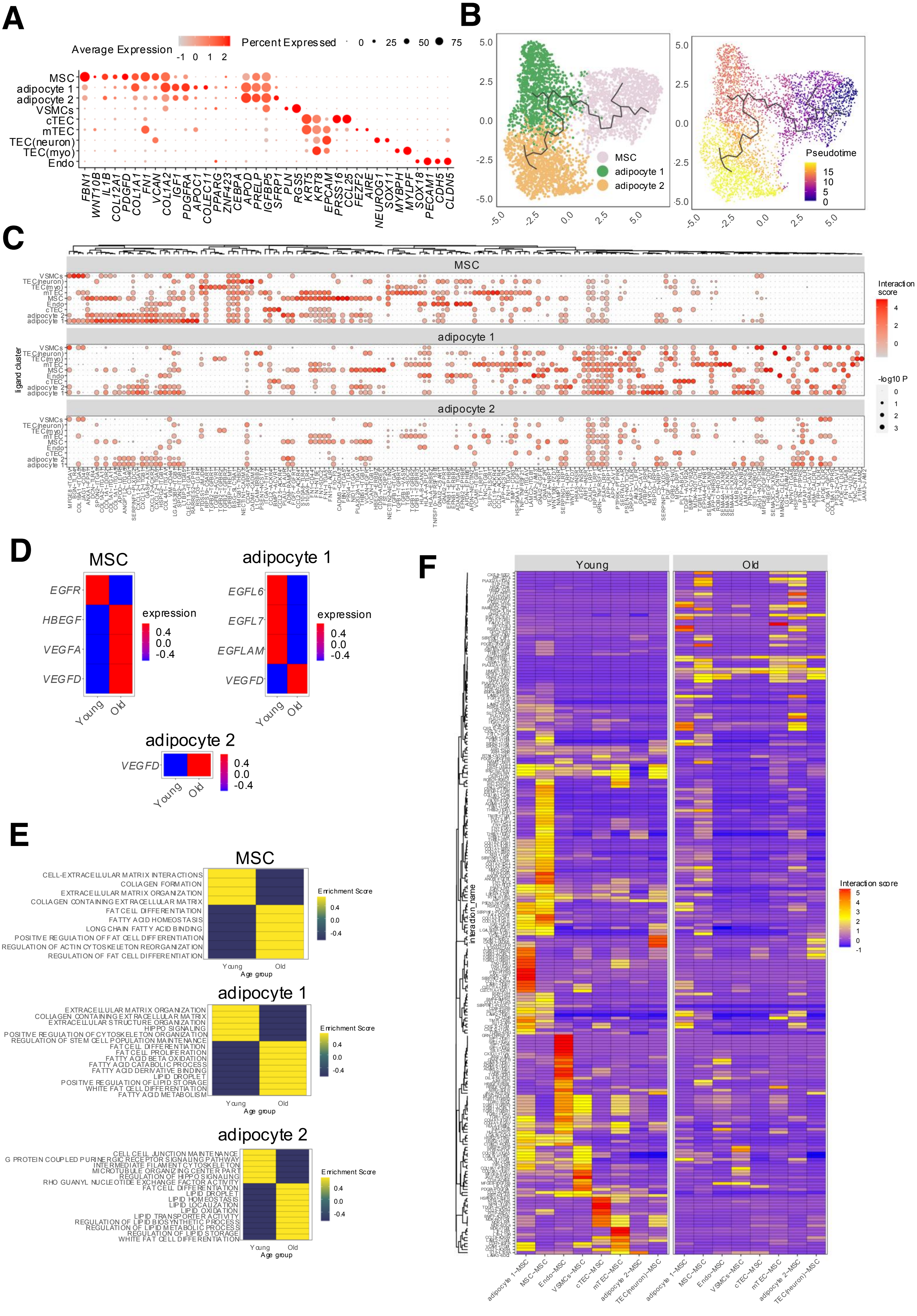

A
Average Expression
-1 0 1 2
Percent Expressed
0 25 50 75
MSC
adipocyte 1
adipocyte 2
VSMCs
cTEC
mTEC
TEC(neuron)
TEC(myo)
Endo
FBN1
WNT10B
IL1B
COL12A1
PDGFD
COL1A1
FN1
VCAN
COL1A2
IGF1
PDGFRA
APOC1
COLEC11
PPARG
ZNF423
CEBPA
APOD
PRELP
IGFBP5
SFRP1
PLN
RGS5
KRT5
KRT8
EPCAM
PRSS16
CCL25
FEZF2
AIRE
NEUROG1
SOX11
MYBPH
MYLPF
SOX18
PECAM1
CDH5
CLDN5
B
MSC
adipocyte 1
adipocyte 2
Pseudotime
15
10
5
0
5.0
2.5
0.0
-2.5
-5.0
C
MSC
adipocyte 1
adipocyte 2
ligand cluster
Interaction score
4
2
0
-log10 P
0
1
2
3
D
MSC
EGFR
HBEGF
VEGFA
VEGFD
expression
0.4
0.0
-0.4
Young
Old
adipocyte 1
EGFL6
EGFL7
EGFLAM
VEGFD
adipocyte 2
VEGFD
E
MSC
CELL-EXTRACELLULAR MATRIX INTERACTIONS
COLLAGEN FORMATION
EXTRACELLULAR MATRIX ORGANIZATION
COLLAGEN CONTAINING EXTRACELLULAR MATRIX
FAT CELL DIFFERENTIATION
FATTY ACID HOMEOSTASIS
LONG CHAIN FATTY ACID BINDING
POSITIVE REGULATION OF FAT CELL DIFFERENTIATION
REGULATION OF ACTIN CYTOSKELETON REORGANIZATION
REGULATION OF FAT CELL DIFFERENTIATION
Enrichment Score
0.4
0.0
-0.4
Young
Old
Age group
adipocyte 1
EXTRACELLULAR MATRIX ORGANIZATION
COLLAGEN CONTAINING EXTRACELLULAR MATRIX
EXTRACELLULAR STRUCTURE ORGANIZATION
HIPPO SIGNALING
POSITIVE REGULATION OF CYTOSKELETON ORGANIZATION
REGULATION OF STEM CELL POPULATION MAINTENANCE
FAT CELL DIFFERENTIATION
FAT CELL PROLIFERATION
FATTY ACID BETA OXIDATION
FATTY ACID CATABOLIC PROCESS
FATTY ACID DERIVATIVE BINDING
LIPID DROPLET
POSITIVE REGULATION OF LIPID STORAGE
WHITE FAT CELL DIFFERENTIATION
FATTY ACID METABOLISM
adipocyte 2
CELL CELL JUNCTION MAINTENANCE
G PROTEIN COUPLED PURINERGIC RECEPTOR SIGNALING PATHWAY
INTERMEDIATE FILAMENT CYTOSKELETON
MICROTUBULE ORGANIZING CENTER PART
REGULATION OF HIPPO SIGNALING
RHO GUANYL NUCLEOTIDE EXCHANGE FACTOR ACTIVITY
FAT CELL DIFFERENTIATION
LIPID DROPLET
LIPID HOMEOSTASIS
LIPID LOCALIZATION
LIPID OXIDATION
LIPID TRANSPORTER ACTIVITY
REGULATION OF LIPID BIOSYNTHETIC PROCESS
REGULATION OF LIPID METABOLIC PROCESS
REGULATION OF LIPID STORAGE
WHITE FAT CELL DIFFERENTIATION
F
Young
Old
interaction_name
Interaction score
5
4
3
2
1
0
-1
adipocyte 1–MSC
MSC–MSC
Endo–MSC
VSMCs–MSC
cTEC–MSC
mTEC–MSC
adipocyte 2–MSC
TEC(neuron)–MSC

Supplementary Fig. 9: Single-cell transcriptomic signature of MSC and adipocyte subgroups and cell-cell interaction analysis in young and old human thymus

(A) Dot plot depicting the DEGs of thymic stromal cells. Dot colors represent the scaled expression and dot size encodes the proportion of gene-expressing cells.

(B) Development trajectory of adipocytes differentiation. Colors represent cell identity (left) or pseudo time (right).

(C) Dot plot showing the cell-cell communication along with the trajectory of adipocytes differentiation. Colors represent interaction intensity and size encodes -log10 *p* value. The cell types in y-axis represent the ligand expressing cells while cell types in facet title represent the receptor expressing cells. Protein pairs involved in cell-cell communication were written in the form of "ligand-receptor" pairs.

(D) Heatmap depicting the expression levels of the EGF-related genes in the subgroups of MSCs and adipocyte at young and old age.

(E) Heatmap depicting the enrichment score calculated by ssGSEA methods indicating the dynamic pathways in MSC and adipocytes during aging.

(F) Cell-cell interactions involving MSCs in young and old human thymus averaged across 13 thymus samples. The cell type labels are written as (cell type expressing the ligand) - (cell type expressing the receptor).

Supplementary Table 1:
Characteristics of the study subjects

Sample	Cell number	Sex	Age group	Health condition
**S1**	2350	M	Old	cardiac surgery
**S2**	648	F	Old	cardiac surgery
**S3**	369	F	Old	cardiac surgery
**S4**	506	M	Old	cardiac surgery
**S5**	252	M	Old	cardiac surgery
**S6**	463	F	Old	cardiac surgery
**S7**	330	F	Old	cardiac surgery
**S8**	223	F	Old	cardiac surgery
**S9**	37	F	Young	cardiac surgery
**S10**	106	M	Young	cardiac surgery
**S11**	153	F	Young	cardiac surgery
**S12**	280	F	Young	cardiac surgery
**S13**	66	F	Young	cardiac surgery

Supplementary Table 2：
Mouse breeding information sheet (2019/11-2023/04)

Genotype	Number	Ratio	Parent
**Homozygote**	18	3.36%	Heterozygous × Heterozygous
**Heterozygote**	345	64.37%	Heterozygous × Heterozygous
**WT**	173	32.27%	Heterozygous × Heterozygous

Supplementary Table 3:

Primers for real-time polymerase chain reaction

Primers specifically designed and oligo synthesized by Genscript (Nanjing, China).

Gene	Forward	Reverse
hMRAP	TTCGTGGTGCTGCTCTTCCTCA	CAGGCACTTCTGGATGCAGAGG
hLPL	CTGCTGGCATTGCAGGAAGTCT	CATCAGGAGAAAGACGACTCGG
hPPARγ	AGCCTGCGAAAGCCTTTTGGTG	GGCTTCACATTCAGCAAACCTGG
hFABP4	ACGAGAGGATGATAAACTGGTGG	GCGAACTTCAGTCCAGGTCAAC
hCD36	CAGGTCAACCTATTGGTCAAGCC	GCCTTCTCATCACCAATGGTCC
hGAPDH	GTCTCCTCTGACTTCAACAGCG	ACCACCCTGTTGCTGTAGCCAA
mMrap	TGTGGTTGAGCCTGGCTACCTT	GGAGGTTGAAGCTGTGAGTCCA
mOcn	GCAATAAGGTAGTGAACAGACTCC	CCATAGATGCGTTTGTAGGCGG
mPPARγ	GTACTGTCGGTTTCAGAAGTGCC	ATCTCCGCCAACAGCTTCTCCT
mCEBPα	GCAAAGCCAAGAAGTCGGTGGA	CCTTCTGTTGCGTCTCCACGTT
mFabp4	TGAAATCACCGCAGACGACAGG	GCTTGTCACCATCTCGTTTTCTC
mDcn	ACTCTCCAGGAACTTCGTGTCC	AGTCCCTGGAAGGCTCCGTTTT
mSca1	GGAGTCTGACTGGAAAGCCGAA	CTTCTGGCAAGCCATCTCCTCA
mPref1	TGGCTGTGTCAATGGAGTCTGC	CCACGCAAGTTCCATTGTTGGC
mCd36	GGACATTGAGATTCTTTTCCTCTG	GCAAAGGCATTGGCTGGAAGAAC
mGpam	GGAAGGTGCTGCTATTCCTG	TGGGATACTGGGGTTGAAAA
mFzd4	CAACCTGTGTGATTGCCTGT	TGTGTGTGGGCTGAAGTGTT
mCmbl	CAAAGCCGTGATTGTCGTCCAG	CCATGGCTCTTGACCCACAAAG
mAngptl4	CCCAACGCCACCCACTTAC	TGAAGTCATCTCACAGTTGACCA
mScp2	CCGGAAAGAGGCAGGTTC	AAAGACGAGGTTTGCCTTGA
mCd302	TCCTCGCTCGTGCTGCTG	GGCACAGCCATCACTGTAAGG
mEtfb	GGTAAAAGTGGAACGGGAAA	TCTCCACCTTGACTCCTGCT
mFoxO1	CTACGAGTGGATGGTGAAGAGC	CCAGTTCCTTCATTCTGCACTCG
mGapdh	CATCACTGCCACCCAGAAGACTG	ATGCCAGTGAGCTTCCCGTTCAG
