# Supplementary material for: MRAP mediated adipocyte differentiation by thymic mesenchymal stromal cells contributes to thymic involution
Source: Nat Commun. 2025 Nov 20;16:10210. doi: 10.1038/s41467-025-64973-z (PMC12635226; doi:10.1038/s41467-025-64973-z)
Supplement: Supplementary file 3 — Peer Review File [file 41467_2025_64973_MOESM3_ESM.pdf]

nature portfolio

Peer Review File

MRAP mediated adipocyte differentiation by thymic mesenchymal stromal cells contributes to thymic involution

Corresponding Author: Dr Wanjun Chen

This manuscript has been previously reviewed at another journal. This document only contains information relating to versions considered at Nature Communications.

This file contains all reviewer reports in order by version, followed by all author rebuttals in order by version.

Version 0:

Reviewer comments:

Reviewer #1

(Remarks to the Author)
The differences in the serum glucocorticoid levels in this mouse model compared to what is reported in both humans and mice that completely lack MRAP is not easily consolidated. However, as the authors state this is a different mouse model and from the data presented where serum glucocorticoids is lower paired with higher levels of serum ACTH this would suggest partial glucocorticoid deficiency. I would suggest that line 403 in the manuscript after reference to supplemetary Fig 5 to add the sentance. "The presence of elevated serum ACTH paired with lower levels of serum corticosterone is suggestive of partial systemic glucocorticoid deficiency". I am satisfied that the authors have aknowledged the limitations due to the absence of tMSC-Mrap specific conditional KO mice. I have no further comments.

Open Access This Peer Review File is licensed under a Creative Commons Attribution 4.0 International License, which

permits use, sharing, adaptation, distribution and reproduction in any medium or format, as long as you give appropriate credit to the original author(s) and the source, provide a link to the Creative Commons license, and indicate if changes were made.
In cases where reviewers are anonymous, credit should be given to 'Anonymous Referee' and the source.
The images or other third party material in this Peer Review File are included in the article's Creative Commons license, unless indicated otherwise in a credit line to the material. If material is not included in the article's Creative Commons license and your intended use is not permitted by statutory regulation or exceeds the permitted use, you will need to obtain permission directly from the copyright holder.
To view a copy of this license, visit https://creativecommons.org/licenses/by/4.0/
